# Mechanistic Insights into the Biological Effects and Antioxidant Activity of Walnut (*Juglans regia* L.) Ellagitannins: A Systematic Review

**DOI:** 10.3390/antiox13080974

**Published:** 2024-08-10

**Authors:** Letiția Mateș, Roxana Banc, Flaviu Andrei Zaharie, Marius Emil Rusu, Daniela-Saveta Popa

**Affiliations:** 1 Department of Toxicology, Faculty of Pharmacy, “Iuliu Hatieganu” University of Medicine and Pharmacy, 6 Louis Pasteur Street, 400349 Cluj-Napoca, Romania; micu.letitia@umfcluj.ro (L.M.); dpopa@umfcluj.ro (D.-S.P.); 2 Department of Bromatology, Hygiene, Nutrition, Faculty of Pharmacy, “Iuliu Hatieganu” University of Medicine and Pharmacy, 6 Louis Pasteur Street, 400349 Cluj-Napoca, Romania; 3 Faculty of Medicine, “Iuliu Hatieganu” University of Medicine and Pharmacy, 8 Victor Babeș Street, 400012 Cluj-Napoca, Romania; zandrei75@yahoo.com; 4 Department of Pharmaceutical Technology and Biopharmaceutics, Faculty of Pharmacy, “Iuliu Hatieganu” University of Medicine and Pharmacy, 12 Ion Creangǎ Street, 400010 Cluj-Napoca, Romania; rusu.marius@umfcluj.ro

**Keywords:** oxidative stress, anti-inflammatory, ellagic acid, urolithins, cardiometabolic health, anti-cancer, biological activity, in vitro, in vivo, clinical studies

## Abstract

Walnuts (*Juglans regia* L.) are an important source of ellagitannins. They have been linked to positive effects on many pathologies, including cardiovascular disorders, neurodegenerative syndromes, and cancer. The limited bioavailability of ellagitannins prevents them from reaching significant circulatory levels, despite their antioxidant, anti-inflammatory, and chemopreventive properties. Urolithins are ellagitannin gut microbiota-derived metabolites. They have better intestinal absorption and may be responsible for the biological activities of ellagitannins. Recent evidence showed that walnut ellagitannins and their metabolites, urolithins, could have positive outcomes for human health. This study aims to synthesize the current literature on the antioxidant activity and mechanistic pathways involved in the therapeutic potential of walnut ellagitannins and their metabolites. In the eligible selected studies (*n* = 31), glansreginin A, pedunculagin, and casuarictin were the most prevalent ellagitannins in walnuts. A total of 15 urolithins, their glucuronides, and sulfate metabolites have been identified in urine, blood, feces, breast milk, and prostate tissue in analyzed samples. Urolithins A and B were associated with antioxidant, anti-inflammatory, cardioprotective, neuroprotective, anticarcinogenic, and anti-aging activities, both in preclinical and clinical studies. Despite the promising results, further well-designed studies are necessary to fully elucidate the mechanisms and confirm the therapeutic potential of these compounds in human health.

## 1. Introduction

Human life and health are constantly impacted by various physical, emotional, or environmental stress-generating conditions, which can increase the levels of reactive oxygen species (ROS) and reactive nitrogen species (RNS) [1]. At higher concentrations, these components may cause oxidative stress (OS) and are risk factors for pathophysiological conditions including lipid oxidation, cardiometabolic disorders (CMD), neurodegenerative diseases, and cancer [2]. Nevertheless, the harmful effects can be alleviated by the presence of antioxidants. These are compounds that, at low concentrations, can delay or prevent OS induction via ROS or RNS exposure [3]. Antioxidants, part of the antioxidant defense system, include endogenous antioxidants, such as superoxide dismutase (SOD), catalase (CAT), glutathione, and glutathione peroxidase, and exogenous antioxidants, vitamins, minerals, and polyphenols [4,5]. Endogenous and exogenous antioxidants play significant roles in the oxidation and antioxidation equilibrium in living systems and act synergistically to maintain redox homeostasis [6].

Polyphenols, which are secondary plant metabolites, have emerged as important preventive and therapeutic bioactive compounds capable of modulating diverse physiological pathways and combating OS and associated health conditions [7]. Tannins, naturally occurring polyphenols found mostly in nuts (walnuts, hazelnuts, almonds, and pistachios) and fruits (pomegranate, berries, grapes, and apples), show significant antioxidant activity [8]. Ellagitannins (ETs), a class of hydrolysable tannins, are esters of hexahydroxydiphenic acid (HHDP) with a polyol, which is usually glucose or quinic acid [9,10]. The HHDP group, a characteristic unit of all ETs, is released by the hydrolysis of ETs with acids or bases and is subsequently spontaneously lactonized into ellagic acid (EA) [10,11]. EA is a dimeric derivative of gallic acid (GA) from which numerous ETs derivatives are formed in plants as a result of the glycosylation, methylation, or methoxylation of its hydroxyl groups [10,12].

The use of ETs is restricted by their limited bioavailability, despite their presence in various natural products [13]. In general, they are large molecules; for example, the molecular weight of Lambertianin D can reach 3740 Da [14]. In addition to their polar nature, the presence of the HHDP moiety in their structure, which is formed by a C-C bond connection between adjacent galloyl residues, results in components with low bioavailability [14]. Most ETs undergo hydrolysis in the gastrointestinal (GI) tract, producing EA [15], which is further converted into urolithins (Uros) under certain physiological conditions by the gut microbiota (GM) [14,16]. Urolithin A (Uro-A) is one of the main metabolites of EA and has demonstrated a variety of bioactivities, including antioxidant, anti-inflammatory, neuroprotective, anti-diabetic, and anticancer effects [17,18]. Additionally, urolithin B (Uro-B) and the isomeric forms, isoUro-A and isoUro-B, can be identified as the final metabolites of ETs and EA [19].

Among dietary plant foods, walnuts (*Juglans regia* L.) are classified as having one of the highest antioxidant activities. Most of the antioxidant activity can be attributed to the polyphenolic constituents, including the ETs, present mainly in the pellicle [20].

Clinical trials have demonstrated that long-term walnut consumption may contribute to cardio-protective effects by lowering OS and inflammation, without concern for adverse effects on body weight or body composition [21]. 

To the best of our knowledge, there are no reviews in the literature that evaluate the antioxidant activity of walnut ETs and their metabolites. We consider such an analysis to be scientifically important, especially since the metabolization of ETs and EA into Uros in the gut and the beneficial health effects of Uros are part of the gut–brain axis [10], which partly explains why the activation of this pathway by walnut consumption is so important to humans. Consequently, this is the first systematic review to analyze the antioxidant activity and preventive or therapeutic potential of ETs and their metabolites after walnut intake and assess the possible mechanisms of action. The evidence found in preclinical and clinical studies and revealed in our systematic review emphasizes that walnut ETs and their metabolites can prevent or reduce the impact of chronic and age-related diseases.

## 2. Methodology

This systematic review was performed following the PRISMA criteria guidelines [22]. The registration code is INPLASY202470086, with DOI 10.37766/inplasy2024.7.0086, https://inplasy.com/?s=INPLASY202470086 (accessed on 22 July 2024).

### 2.1. Eligibility Criteria

Our systematic review included (1) studies performed on both peeled and unpeeled walnut kernels, as well as on walnut pellicle plant material; (2) the identification and/or quantification of EA, ETs, and their active metabolites/Uros; and (3) the examination of the biological activity of identified compounds via (4) in vivo testing, (5) in vitro testing, and (6) in clinical studies. 

We excluded (1) abstracts, narrative reviews, comments, opinions, methodological papers, editorials, conference abstracts, or any other publications lacking primary data and/or explicit methodological explanations; (2) publications with the full text not available; (3) duplicate studies or databases; and (4) publications in languages that were not known.

### 2.2. Information Sources

We performed a systematic literature search in PubMed, EMBASE, Scopus, and ClinicalTrials.gov databases for studies describing the identification and/or quantification and the biological activity of EA and ETs from walnut (*J. regia*) and their active metabolites. We conducted the search from the inception of each database through to 31 May 2024. The literature search had no language constraints. We also screened the bibliographies of the included studies and current reviews to ensure the thoroughness of the research.

### 2.3. Search Strategy

To search the databases, we used a combination of free-text words, along with their synonyms, singular and plural forms, and thesaurus words (Medical Subject Headings for PubMed: ((juglans[MeSH Terms]) OR (juglans[Title/Abstract]) OR (walnut[MeSH Terms]) OR (walnut[Title/Abstract]) OR (walnuts[Title/Abstract]) OR (juglans regia[MeSH Terms]) OR (juglans regia[Title/Abstract])) AND ((ellagic acid[MeSH Terms]) OR (ellagic acid[Title/Abstract]) OR (ellagitannin[Title/Abstract]) OR (ellagitannins[Title/Abstract]) OR (urolithin[Title/Abstract]) OR (urolithins[Title/Abstract])), Emtree for EMBASE: (‘juglans’/exp OR ‘juglans’ OR ‘walnut’/exp OR ‘walnut’ OR ‘walnuts’ OR ‘juglans regia’/exp OR ‘juglans regia’) AND (‘ellagic acid’ OR ‘ellagitannin’ OR ‘ellagitannins’ OR ‘urolithin’/exp OR ‘urolithin’ OR ‘urolithins’) and Scopus: (TITLE-ABS-KEY (juglans) OR TITLE-ABS-KEY (walnut) OR TITLE-ABS-KEY (walnuts) OR TITLE-ABS-KEY (juglans AND regia) AND TITLE-ABS-KEY (ellagic acid) OR TITLE-ABS-KEY (ellagitannin) OR TITLE-ABS-KEY (ellagitannins) OR TITLE-ABS-KEY (urolithin) AND TITLE-ABS-KEY (urolithin)).

### 2.4. Selection Process

Three investigators (L.M., F.A.Z., and R.B.) conducted a comprehensive examination of the titles and abstracts to identify the articles with relevance to this study. Subsequently, the whole texts of the documents that appeared to meet the selection criteria were obtained for further evaluation. Each whole text was independently checked by the same investigators. In the event of a disagreement, the studies were debated until a consensus was achieved. Only the most recent or useful article was chosen where there were multiple publications from the same trial.

### 2.5. Data Items

Data regarding the outcomes were extracted in a spreadsheet Microsoft (Microsoft Office 365, MS, Redmond, WA, USA) Excel file containing the following data: materials/type of extract/biological samples, phytochemical composition studies, in vitro studies/biological systems analysis methods, in vivo studies/animal models, and clinical trials/metabolites profile and their biological effects. Furthermore, data regarding study characteristics were extracted in a spreadsheet file: country, study design, study purpose, and study outcomes. Other investigators than those who extracted the initial full-text articles rechecked the extracted data (D.-S.P. and M.E.R.).

**Figure 1:**
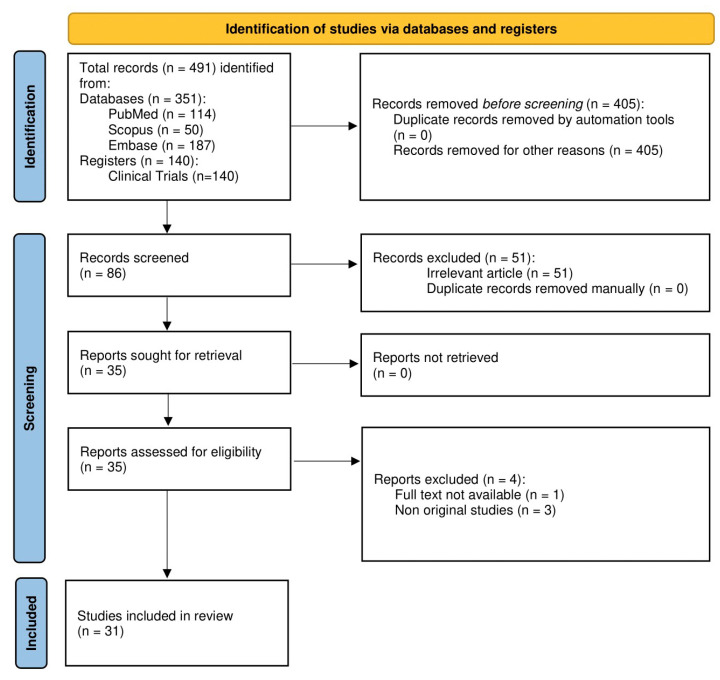
PRISMA flow diagram of study selection.

A total of 86 articles were considered from the systematic search and review of relevant reference lists. After applying exclusion criteria, 31 articles were included in the systematic review. The procedure of study inclusion and exclusion is shown in Figure 1. The characteristics of the included studies [9,11,23,24,25,26,27,28,29,30,31,32,33,34,35,36,37,38,39,40,41,42,43,44,45,46,47,48,49,50,51] are revealed in Table 1.

## 3. Results and Discussion

### 3.1. Phytochemical Composition

The publications included in this study indicated that the beneficial effects of walnuts, which include a wide range of biological activities, can be related to the presence of several bioactive compounds. 

Among the articles selected for the present review, 61.29% of the studies examined the phytochemical composition of the peeled or unpeeled kernel and/or pellicle of the walnut (*n* = 19). The main compounds responsible for the biological activities of walnuts, i.e., phenolic constituents, including hydrolysable tannins—ETs and gallotannins, condensed tannins, and phenolic acids—and their derivatives; flavonoids; anthocyanins; phenolic aldehydes; stilbenes; naphthoquinone; coumarins; and dicarboxylic acid derivatives, were investigated in all phytochemistry studies (*n* = 19). Only 19.35% of the studies examined the other types of non-phenolic compounds present in walnuts, including sesquiterpenoids, biogenic monoamines, amino acids, polypeptides, amides, di- and polysaccharides, alkaloids, and carboxylic acids (*n* = 6).

According to the findings, the composition of the phytochemicals found in walnuts showed variations, both quantitatively and qualitatively, between the kernel and the pellicle within the same cultivar. These were also seen within the same type of plant material when originating from different cultivars.

ETs and EA are among the most potent antioxidant constituents of walnuts. The findings regarding ETs and EA derivatives in the kernels and pellicles of walnuts [9,11,23,24,26,27,28,29,31,35,38,39,40,41,43,44,47,48,50] are summarized in Table 2.

#### 3.1.1. Walnut Sample Preparation and ETs and EA Extraction

For the investigation of the phenolic profiles, it was essential to consider the preparation of the plant material before the analysis. Due to the intricate nature of the samples, the outcomes of the entire extraction process were significantly influenced by the methods used for sample preparation. 

Standard sample preparation methods, such as drying [9,26,41], grinding [26,27,28], and lyophilization [43], were commonly used prior to the extraction process. Nut samples were either air-dried away from sunlight or dried in driers with a circulating air flow at 30–35 °C to a moisture content of 6–8% [9,26,41]. In order to grind the nut kernel and pellicle to a fine texture—either crushing the plant material in a mortar with a pestle or grinding it in a mill—a mechanical grinder or blender was used [9,11,23,26,28,48]. Also, before the extraction of the phenolic compounds, the walnut kernel samples (ground to a fine texture/powder) were subjected to degreasing with non-polar solvents such as hexane [27,28,48], cyclohexane [29], or petroleum ether [41,44,47,50].

As a result of the complex composition and nature of the complexes that form with both polysaccharides and proteins, there is no universally applicable extraction technique that is suitable for the extraction of all the phenolic compounds found in walnuts. Thus, in the reviewed studies, various techniques and solvents were employed to extract ETs and EA derivatives from either the kernel or the outer skin of the walnut. The most frequently used extraction methods were solid–liquid extraction (*n* = 7) and sonication (*n* = 5), followed by vortexing and sonication on an ice bed/in ice water/in a cold-water bath (*n* = 3), ultra-turrax homogenization (*n* = 1), homogenization (*n* = 1), and stirring (*n* = 1), respectively. 

Solid–liquid extraction is the simplest and most traditional method used for the extraction of phenolics, including tannins. Thus, through the direct contact of the solid plant material with the solvent, this technique allows for the separation of soluble compounds, without the need for the additional mechanical treatment of the samples [14].

The main parameters influencing the solid–liquid extraction process, which are also considered in the studies contained in this review, are the nature of the solvent, the extraction temperature, the extraction time, and the solid–liquid ratio. 

The nature of the solvent and its polarity are of significant importance. The solubility of phenolic compounds in the extraction solvent is the primary determinant of polyphenol recovery from plant materials. In general, methanol, ethanol, acetone, and their aqueous mixtures are often used as organic solvents to extract phenolic compounds from edible plant parts [41,52]. In terms of polarity, solvents can be classified according to their dielectric constant as follows: acetone (20.7) < ethanol (24.3) < methanol (32.6) < water (78.43) [53,54].

Extraction yields increase with solvent polarity. High extraction yields are generally obtained using methanol and ethanol and their mixtures with water, but they are also obtained with other widely used solvents, such as acetone or ethyl acetate, while a poor extraction efficiency is obtained with hexane [14,52,55]. This is explainable because hexane, an apolar solvent, has a reduced ability to solubilize polar phenolic compounds. Most of the studies included in this review used aqueous methanol as the extraction solvent in varying proportions (*n* = 6). Malik et al. [56] used 80% aqueous methanol to extract EA and its derivatives from pecan kernels. The extraction solvents often used in other studies are aqueous ethanol (*n* = 5), acetone in combination with water (*n* = 4), and methanol (*n* = 4). The results obtained by Wu et al. [41] showed that 70% aqueous methanol extractions obtained higher values of hydroxycinnamic acids and hydroxybenzoic acid in free form, followed by aqueous ethanol and aqueous acetone. Also, 70% methanol showed the best extraction capacity for bound insoluble phenolics, while 70% acetone extraction led to a significantly higher content of soluble phenolics (free and esters), including EA, than methanol and ethanol extracts [41]. In the study of Vijayalaxmi et al. [52], the extraction of phenolic compounds from peanut husk was carried out using solvents of different polarities: 50%, 70%, and 100% methanol; 50%, 70%, and 100% ethanol; and water. Among all the solvents studied, 50% methanol proved to be the most suitable solvent for the extraction of polyphenols compared to the different concentrations of ethanol, leading to the highest content of total polyphenols, total tannins, and flavonoids, but also to the highest extraction yield.

Other parameters, such as extraction time, extraction temperature, and the solid–liquid ratio, can have a major influence on the results. Increasing the extraction time can ensure a higher content of extracted tannins due to longer contact times between the solute and the solvent. However, increasing the duration and time of extraction above certain values can slow down the extraction by creating an equilibrium between the concentration of tannins from the plant matrix and the solvent in the case of an increase in the first parameter, and, respectively, lead to the denaturation of tannins in the case of high temperatures. Regarding the solid–liquid ratio, it was reported that with the decrease in this parameter and the use of higher amounts of solvent, higher extraction yields were obtained [55]. In the studies in this review, solid–liquid extraction was performed at room temperature or at 40 °C for 2 or 6 h, and the solid–liquid ratio varied widely between studies, ranging from 1:5 to 1:50 [27,41,43,47].

The ultrasound-assisted extraction method is an unconventional extraction technique. It is simple, cheap, and with the advantage of short extraction times. The mechanical vibrations caused in the plant matrix by the bubbles induced by the sound waves can break the cell wall tissues, improving the penetration of the solvent into the matrix and leading to larger amounts of extracted tannins being obtained. The extraction yield of active substances depends on variables such as sonication time and power, the solvent selected for extraction, and the temperature of the water bath [14,55]. 

As in the case of solid–liquid extraction, the use of more polar solvents leads to higher extraction yields, with a higher selectivity of solvents such as ethanol and methanol being reported for polyphenolic compounds with higher amounts of tannins in the extracts [55]. Thus, in the present review, the solvent most often used in the case of ultrasonic extraction was methanol or 50% aqueous methanol (*n* = 4), followed by 60% or 70% aqueous acetone (*n* = 3), and 80% aqueous ethanol (*n* = 1). Solvents have also been reported to affect the composition of extracts [57]. Thus, when methanol was used as a solvent, phenolic acids were predominant in the walnut kernels [26], while in the water/acetone extracts the most abundant were ETs, EA, and their derivatives [11]. Therefore, the superior ability of aqueous acetone to extract ETs from walnut and its derivatives (walnut pellicle) is noteworthy.

In terms of extraction times, good extraction yields have been reported at sonication intervals below 1 h. Extending the duration of sonication may lead to a decrease in tannins and their possible degradation [55]. Also, increasing the sonication power above a certain point results in a decrease in extracted tannins due to their chemical decomposition [58]. For the studies included in this review, the sonication time was shorter (from 1 min or 5 min, respectively, to 30 or 40 min, respectively) when the water bath temperature was higher (30 °C or 40 °C) [23,26,28,39,48], while in the case of sonication in ice, the sonication duration was sometimes extended up to 1 h [9,50]. Other authors also confirmed that a longer sonication time can increase the extraction yield for polar compounds in hydro-alcoholic solvents [59,60], while a prolonged exposure to higher temperatures can significantly decrease the recovery of phenolic compounds due to their thermal degradation [59].

Homogenization, another extraction method also used by some studies in this review, used 80% aqueous methanol as the solvent [24,39]. 

#### 3.1.2. Separation and Characterization of Walnut ETs and EA

The extracts obtained by utilizing the previously discussed extraction processes were intricate mixtures that required separation due to their diverse natural components and impurities. Separation is a purification method that is frequently employed in conjunction with characterization techniques to identify various molecules [12].

The process of purifying compounds using macroporous resin was addressed in the study by An et al. [47]. After passing the extract through the polar macroporous adsorbent resin, the adsorbed materials were washed with water to remove water-soluble impurities (sugar, proteins) and then eluted with mixtures of ethanol and water in different proportions. Purification with macroporous resins has also been reported for the purification of flavonoids from *Platycladus orientalis* (L.) Franco, a nutritious traditional food plant [61].

Identifying, separating, and quantifying ETs is challenging because of their complicated structure/high molecular weight, high polarity, and, in some instances, the absence of commercial standards [14]. Polymers such as ETs can be hydrolyzed with acids or bases to produce EA, which can be used to indirectly quantify ETs [24]. Some of the studies examined in this review used acid hydrolysis (2 M HCl, heating at 85 °C for 20 h) for the analysis of ETs, with their quantification as EA equivalents [24,31,38].

High-performance liquid chromatography (HPLC) and ultra-performance liquid chromatography (UPLC) were the most used techniques for the separation and isolation of ETs and EA. Most often, reversed-phase HPLC (RP-HPLC), performed on a C18 column, was used with polar mobile phases, such as water containing 0.1–1% formic acid/2% acetic acid/0.3% trifluoroacetic acid; methanol or acetonitrile (acidified or not with formic acid); or a mixture thereof [9,11,23,24,27,35,41,43,47,48,50]. 

In terms of detection, for the identification and quantification of ETs and EA, UV detectors, such as diode array detectors (DAD/PDA) were employed in many of the studies examined in this review [23,24,26,27,29,31,35,38]. UV chromatograms of extracts were recorded at 252, 255, 280, and 360 nm. ETs and EA presented characteristic UV spectra with maximum absorbance values below 270 nm [14]. Mass spectrometry (MS) detectors are also used extensively for the identification of ETs and EA, often in combination with HPLC-DAD systems, and the electrospray ionization (ESI) source is operated in the negative mode [9,11,24,27,31,38,39,41,43,48,50]. The tandem use of HPLC-DAD and ESI-MS is a technique that has been applied by many researchers to identify and quantify ETs and EA, both in walnuts [62] but also in other plant matrices, such as pomegranate [63] or *Anogeissus leiocarpus* stem bark [64]. 

While MS is the primary analytical technique used in the majority of research aiming at chemical identification in mixtures, nuclear magnetic resonance (NMR) offers complementary advances that warrant investigation for achieving improved structural confidence [65]. Thus, for the complete characterization of ET profiles from walnut kernels, Harammishi et al. [39] studied the phytochemical composition of extracts using LC-HR-ESI-MS/MS and NMR, identifying three new ETs based on UV, 1H-, and 13C-NMR spectra. NMR also proved a valuable tool when used in conjunction with HPLC-DAD-MS analysis for the identification of 43 ETs in *Anogeissus leiocarpus* stem bark [64].

Capillary electrophoresis (CE) was used in a single study in this review to determine ETs and EA derivatives [28]. An uncoated fused silica capillary of 90 cm length was used and the optimal condition for EC separation was obtained by using 40 mM ammonium acetate (pH 9.5) as running buffer. The CE system was coupled to a microTOF-MS. CE is a relatively new separation method that has the potential to become a more widely used method for the characterization of phenolic compounds and to serve as an alternative to HPLC [66]. It was also used by other authors for the determination of EA in Argentinian wines [67].

#### 3.1.3. Walnut ETs and EA Derivatives

EA and its derivatives, as well as ETs, have been characterized in the kernels and pellicles of walnuts. In total, 23 EA derivatives and 62 ETs and isomers were described in the eligible studies. Of the analyzed studies, over 58% identified EA and EA derivatives (*n* = 18), while ETs were found in 35.48% (*n* = 11) of the studies.

In quantitative analyses, EA was detected in most of the phenolic composition studies (*n* = 9), compared to its derivatives (*n* = 2) and ETs (*n* = 2), respectively. Significant amounts of EA were reported in the *J. regia* walnut, both in the kernel but especially in the pellicle. Thus, EA was found by Colaric et al. [26] to be one of the most abundant phenolic compounds in the walnut, after syringic acid and juglone, with the walnut pellicle having an average 21.3 times higher EA content (128.98 mg/100 g) than the walnut kernel (5.90 mg/100 g). 

A previous study conducted by Slatnar et al. [68] confirmed the uneven distribution of phenolic compounds in walnuts between the kernel and the skin. Thus, walnut pellets, a waste product obtained from the pressing of walnut oil, consisting mainly of kernel skins, showed a significantly higher content of EA and derivatives than the walnut kernel. 

The abundance of EA in the skin covering the walnut kernel was also confirmed in the study by Mahoney and Molyneux [23], where EA contributed up to 15.9% of the dry weight of the pellicle, compared to GA, which contributed up to only 3.4%. In contrast to *J. regia*, in two black walnut species (*J. hindsii* and *J. nigra*), the contribution of EA to the dry weight of the pellicle was much lower, standing at only 3.1% and 2.6%, respectively.

Regarding the variations in EA content, statistically significant differences (*p* < 0.05) were observed between ten different varieties, both in the walnut kernel (3.26–9.66 mg/100 g) and in the pellicle (60.66–266.19 mg/100 g) [26]. Another study reported the Chandler variety to be the richest in terms of EA (24.7 mg/kg dw), with amounts in the kernel two times higher than those of the Howard variety (12.4 mg/kg dw) and four times higher than those of the Hartley variety (6.9 mg/kg dw), respectively [28]. 

Most of the studies that reported the content of phenolic antioxidants in the walnut focused on the free soluble form of these compounds [9,26,35,68]. However, these compounds, including EA and ETs, can also be found in the food matrix as soluble esters or conjugated and insoluble bound forms. These have demonstrated important health-promoting effects, including exceptional bioactivity, and are released from the food matrix during food processing [49].

Thus, Wu et al. [50] showed that EA represented more than 20%, 40% and 15% of the antioxidants in the kernel in free, esterified, and bound forms, respectively, being the most significant contributor to antioxidant activity in all three forms (free, esterified, and bound). Therefore, EA could be considered a unique feature of walnut compared to other nuts. EA was found to be the most abundant component. This was the case in not only the kernel (62.9%) but also in the skin (69.0%), where it was found to be the most powerful phenolic antioxidant among the bound phenolics of the pellicle [50].

ETs and EA are covalently bonded to the structural components of the cell wall in their insoluble forms. They form ether linkages with lignin through their aromatic ring hydroxyl groups and ester linkages with structural carbohydrates and proteins through their carboxyl groups [69]. 

While the consumption of bound phenolics has a positive impact on the prevention of colon cancer, the dietary intake of free and soluble conjugated forms has demonstrated numerous other health benefits [10,69]. In this regard, potential approaches to transforming these insoluble bound forms into more bioactive fragments include the use of heat, enzymes, fermentation microorganisms, and alkali or acid cooking [69]. Thus, an increase in free EA content was also obtained after the baking process, when the ETs from the walnut kernel were hydrolyzed, releasing polyol (mainly glucose) and HHDP groups, from which EA was subsequently produced [48]. Given that EA is considered an antioxidant with multiple functions, demonstrating numerous health benefits in vitro, including anti-inflammatory, anti-cancer, cardioprotective, and neuroprotective properties, as well as the inhibition of OS, its release after baking could promote the production of Uros, the main bioactive forms responsible for beneficial effects in vivo [10,48].

Among the ETs, pedunculagin was detected in most of the phenolic composition studies (*n* = 10), followed by casuarictin and strictinin (*n* = 8), casuariin, isostrictinin, and tellimagrandin I (*n* = 7), casuarinin and glansreginins A and B (*n* = 6), glansrin C (*n* = 5), and HHDP-glucose and valoneic acid dilactone (*n* = 4). Additionally, 50 ET derivatives were detected less often in the studies included in this review (*n* = 1–3).

Regarding the quantitative analysis, glansreginin (Gla) A was found to be the most abundant compound in the peeled walnut kernel, followed by Gla B. However, there were great variations between the cultivars, with the Fernette cultivar having a Gla A content (846.7 mg/kg fresh weight (fw)) 8.22 times higher than the Sava cultivar (103.0 mg/kg fw), while the Fernor cultivar (175.8 mg/kg fw) exceeded the Gla B content of the Krka cultivar (84.1 mg/kg fw) by more than two times [9]. In the study by Gomez-Caravaca et al. [28], Gla A and Gla B were also quantitatively predominant in the unpeeled walnut kernels, with the Howard variety having the highest amount of Gla A (335.6 mg/kg dry weight (dw)), but the lowest amount of Gla B (35.5 mg/kg dw), while the Hartley variety presented the highest content of Gla B (99.9 mg/kg dw). 

In a previous experiment, in which Gla A was also reported to be the most abundant compound in walnut kernels, very similar content results were obtained in the Fernette (817.31 mg/kg bw), Fernor (372 mg/kg bw), and Franquette (328.67 mg/kg bw) cultivars to those reported by Medic et al. [9] for the same varieties (846.7 mg/kg fw; 336.2 mg/kg fw; and 315.0 mg/kg fw, respectively) [68].

In contrast to the results obtained by Medic et al. [9] for walnut kernels, in a subsequent study carried out on chestnuts, the content of Gla B was up to 10 times lower compared to that in the peeled walnut kernel, while Gla A, the most abundant compound in peeled walnut kernels, was not even found in trace amounts in chestnuts [70].

In order to identify the characteristic components of walnuts, Haramiishi et al. [39] compared walnut kernel extracts with the extracts of six other nuts: almond, pistachio, hazelnut, cashew, pecan, and macadamia. Thus, Gla A, with the main peak being in the walnut extract, could be an indicator component of walnut quality as this peak was not observed in any other nut extract. However, previous studies reported the presence of Gla A in pecan (*Carya illinoinensis*) [71] and hazelnut (*Corylus avellana*) kernels, but much lower levels of Gla A (18.00–110.92 mk/kg dw) and Gla B (9.59–56.12 mk/kg dw) were found in hazelnut kernels [72] compared to walnut kernels (76.3–335.6 mk/kg dw and 35.5–99.7 mk/kg dw, respectively) [28]. Similarly, the results of a recent study showed lower levels of Gla A (6.8–47.0 mg/kg dw) in kernels from 12 cultivars of black walnut (*J. nigra*) compared to English walnut (*J. regia*), confirming that Gla A is only the most abundant compound in *J. regia* walnut kernels, but not in the kernels of other nuts [73].

Pedunculagin, a main ET from walnuts with a wide range of antioxidant and anti-inflammatory properties [74], was not quantified in the walnut kernels, but only in the walnut skin (3.1–13.3 mg/g fw) [9].

A single study selected for this review carried out a quantitative analysis of ETs in the pellicle of *J. regia*. Thus, the most abundant compounds in the walnut pellicles from six different cultivars (Fernor, Fernette, Franquette, Sava, Krka, and Rubina) were casuarin/casuarictin isomer 1 (23.9–39.8 mg/g fw), castalagin/vescalagin isomer 2 (22.1–35.9 mg/g fw), and tellimagrandin I isomer 3 (18.4–27.9 mg/g fw) [9]. Contrary to these results, Slatnar et al. [68], working with the six isomers of vescalagin, detected lower contents in the walnut pellets of Fernette (17.90–111.34 mg/kg fw), Fernor (64.34–356.63 mg/kg fw), and Franquette (20.60–196.62 mg/kg fw) varieties, compared to those obtained by Medic et al. [9] for the same cultivars (9.5–17.8 mg/g fw; 11.7–30.6 mg/g fw; and 15.0–35.9 mg/g fw, respectively). The differences could be explained by the fact that polyphenols in general, including ETs, EA, and derivatives, are secondary plant metabolites that are biosynthesized under stress conditions, having a defensive role [75]. The content of these compounds in walnuts can vary significantly from year to year depending on environmental factors (geoclimatic factors—temperature, humidity, UV radiation, soil composition, etc.), but also on fruits’ ripeness and harvest time [76]. 

### 3.2. Metabolism of Walnut ETs and EA

In vitro human GM fermentation models are considered valid tests to explore the mechanisms in the sites of metabolism pathways. The complementary approaches, which include both in vitro and in vivo models, offer strong outcomes in terms of values [77].

#### 3.2.1. Urolithin Biosynthesis from ETs and EA Metabolism

ETs undergo a metabolism process in the upper GI tract, followed by lactonization, as a result of their susceptibility to hydrolysis in the acidic conditions of the stomach and the slightly alkaline environment of the small intestine [78]. This process results in the synthesis of EA [79].

Current research has shown that walnut seeds contain significant amounts of ETs and EA, both in the kernel [11,31,39,43,47,48] and the pellicles [9]. The most relevant ETs identified and quantified in walnuts are pedunculagin, casuarictin, and Gla A [9,28]. Humans metabolize EA and ETs to dibenzopyran-6-one derivatives known as Uros. Cerda et al. [25] demonstrated that Uros are metabolites of microbial origin.

Indeed, in vitro bioavailability studies have shown that acidic conditions (HCl, pH 1.8–2.0) and gastric enzymes do not have the ability to hydrolyze ETs [80]. The release of EA from ETs occurs in the small intestine under specific physiological conditions, primarily due to the pH levels (7.0–7.3) rather than the influence of bile salts and pancreatic enzymes [81]. Most ETs undergo acidic or basic hydrolysis in the GI tract, which results in the formation of EA via the hydrolyzation of the ester bonds of ETs. This process is facilitated by the enzyme ellagitannin acyl hydrolase [15]. Nevertheless, ETs that fail to hydrolyze remain intact in the large intestine [78]. EA, like ETs, has a low bioavailability due to its poor water solubility and its ability to bind irreversibly to cellular DNA and serum proteins, processes that result in complexes of large molecular weight that are unable to permeate cell membranes [82]. In contrast, EA is converted into Uros by the GM under physiological conditions [14].

The urolithin biosynthesis pathway in humans has been investigated and documented by Tomas-Barberan et al. [83] in fecal fermentation samples and a GI simulator model (TWIN-SHIME). 

**Figure 2:**
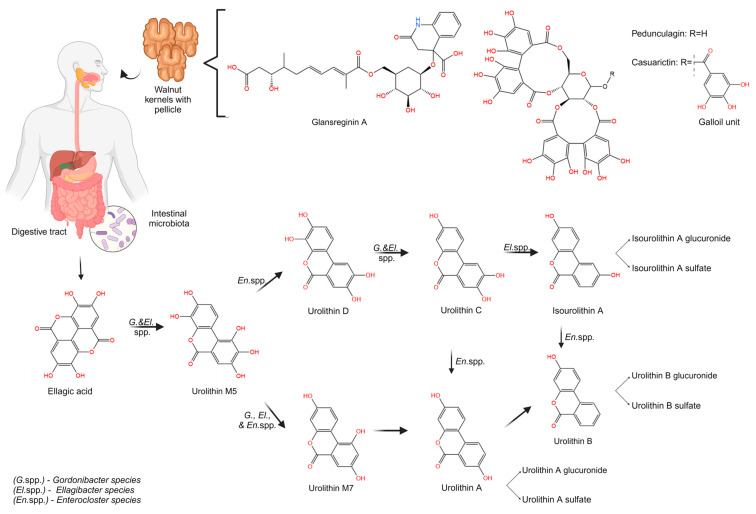
The main metabolites of ETs and EA formed after walnut *(J. regia* L.) intake by intestinal microbiota (created with BioRender.com).

The catabolic pathway of ETs and EA to Uros is represented in Figure 2.

The formation of Uros occurs when one of the lactone rings in EA opens and decarboxylates, subsequently leading to the sequential and gradual removal of hydroxyl groups from various locations through dehydroxylase activities [19]. The first step in the urolithin metabolite pathway is the hydrolysis of the lactone ring in EA, which leads to luteic acid synthesis, followed by decarboxylation and dihydroxylation processes and the formation of pentahydroxy-urolithin (Uro-M5), an important intermediate product [14,16]. The GM can transform EA by cleaving the lactone ring, as well as by decarboxylation and dehydroxylation processes, starting with Uro-M5. Further, in this regard, Uro-M5 is converted into tetrahydroxy-urolithins, specifically Uro-D, Uro-M6, and Uro-E, by the enzymatic activity of 10-, 4-, and 9-dehydroxylases, respectively [14]. The dehydroxylase enzymes continue this process, producing trihydroxy-urolithins (Uro-C and Uro-M7), which ultimately generate dihydroxy-urolithins (Uro-A and iso-Uro-A) and monohydroxy-urolithins (Uro-B), which are typically detectable in the examined biological samples [16,84].

#### 3.2.2. Influence of Individual Metabotype and GM on Urolithin Biosynthesis

The metabolism of ETs and EA, as well as the synthesis of Uros, varies significantly among individuals [14]. This variation is influenced by the composition of the GM, which, in turn, is affected by factors such as the individual’s health, age, environmental and life conditions, and human urolithin metabotype (UM) [33,85].

Currently, humans are understood to possess three urolithin-producing metabotypes that are associated with their capacity to produce Uros [14,86]. Individuals who exclusively produce Uro-A conjugates are classified as “Metabotype A” (UM-A), while those who produce isoUro-A and/or Uro-B in addition to Uro-A are classified as “Metabotype B” (UM-B). “Metabotype 0” (UM-0) individuals are undetectable for any of these Uros [84,86]. Indeed, according to Cortés-Martín et al. [85], the population between the ages of 5 and 30 is divided into approximately 70% UM-A and 20% UM-B types. These two metabotypes are typically associated with a decrease in UM-A and an increase in UM-B in individuals over the age of 30, while the percentage of UM-0 remains at approximately 10%. Nevertheless, longitudinal studies need to be conducted to assess the stability of the UM over time in a specific individual [86].

The microbiota is a biological community of symbiotic, commensal, and pathogenic microorganisms that collectively inhabit our body space [87]. The main ways it influences human health are modulating inflammation; digesting nutrients from the diet; and promoting gut integrity, glucose and lipid metabolism, and immune responses [88]. Microorganisms in the intestine are connected to polyphenols that are not readily absorbed by the body through a complex two-way relationship [17]. According to D’Amico et al. [17], EA has a beneficial impact on the GM by promoting the proliferation of beneficial bacteria and suppressing harmful ones. Consequently, certain gut bacteria convert EA into Uros that are both readily absorbed and beneficial, particularly in the context of enhancing metabolic and cardiovascular health [16].

The presence of *Gordonibacter urolithinfaciens* and *Gordonibacter pamelaeae* plays a significant role in converting EA and ETs into Uros. The *Gordonibacter* and *Ellagibacter* genera, which are members of the *Coriobacteriaceae* and *Eggerthellaceae* families, respectively, are the primary GI bacteria that are known to characterize various metabotypes by converting EA into Uros through lactone-ring cleavage, decarboxylation, and catechol dehydroxylations at 4- and 10-positions [14]. EA is metabolized by the genus *Gordonibacter* into Uro-M5, Uro-M6, and Uro-C, while *Ellagibacter* can convert EA into Uro-M5, Uro-M6, Uro-C, and isoUro-A. However, both genera can convert Uro-D and Uro-M6 into Uro-C [84]. *Enterocloster bolteae*, a novel bacterium in the *Lachnospiraceae* family, was isolated from human feces. This bacterium can convert Uro-C and isoUro-A into Uro-A and Uro-B, respectively [84]. Subsequently, the urolithin formation profiles associated with metabotype A and B individuals were cooperatively reproduced by co-cultures of these bacteria, which possess complementary activities during in vitro fermentation [84] (Figure 2).

#### 3.2.3. Bioavailability of Urolithins and Their Metabolites after Walnut Intake

Uros exhibit a higher absorption rate compared to ETs and EA, attributed to their enhanced fat solubility, which is one of the relevant factors for this characteristic [83]. After absorption in the colon, Uros enter the enterohepatic circuit and undergo significant phase-II metabolism to produce sulphates and glucuronides. The urolithin conjugates may be identified in plasma and systemic tissues at quantities ranging from low micromolar (µM) to high nanomolar (nM) levels [89], even for a period of 3–4 days after absorption [90].

So far, a total of 15 Uros and their associated metabolites (glucuronides and sulfates) have been identified in different human fluids and tissues, including urine, blood, feces, breast milk, and prostate samples [16,31,38]. As previously mentioned, the capacity to produce Uros, and therefore induce the health effects related to ET consumption, differs within individuals due to variations in their gut flora, as not all persons possess the required bacteria to produce all Uros [84,91]. After consuming several sources of ETs, human plasma has been discovered to include methylated and glucuronidated analogues such as urolithin-A glucuronide (Uro-A-gluc), urolithin-C glucuronide (Uro-C-gluc), urolithin-C methyl ether glucuronide, and dimethyl ellagic acid glucuronide [31].

In animals, the presence of urolithin conjugates in plasma and urine samples has been extensively investigated. Rodents (rats and mice), ruminants, pigs, and other mammals were the primary animals incorporated into these investigations [14]. Moreover, following the consumption of ET-rich foods, such as walnuts, the levels of Uro-A and Uro-B conjugates were measured in plasma and urine samples taken from humans [25,31,34,37,38]. The primary urolithin conjugates were detected at micromolar concentrations in human plasma (Uro-A gluc up to 35 µM, Uro-B gluc 7.3 µM, and isoUro-A 0.745 µM, respectively) [83]. In an update of urolithin concentrations in different biological fluids after consuming different sources of ETs, García-Villalba et al. [16] found up to 50 μM in urine samples. The high variability was attributed to the diet source of ETs, the administered dose, and the analytical procedures employed. In addition, small quantities of Uro-A gluc and Uro-B gluc were detected in human prostate samples after the intake of walnuts or pomegranate juice [31].

#### 3.2.4. Pharmacokinetics of Uro-A and Uro-B after Walnut Intake

Uro A and Uro B, which are produced at the intestinal level, are absorbed into systemic circulation and distributed at the tissue level. Further, they may undergo biotransformation reactions such as glucuronidation, sulfatation, or methylation. Conjugation processes, involving glucuronic acid or sulfate ions, may occur in the intestine, enterocytes, and liver [17]. The primary metabolites detected in human plasma and urine samples following the consumption of foods containing ETs are Uros A and B, their glucuronides (Uro A-gluc and Uro B-gluc), and their sulfate conjugates (Uro-A sulfate and Uro-B sulfate) [16,33,34]. Nevertheless, the excretion levels of various forms of Uros and their metabolites vary significantly among individuals, depending on the UM [83,84,86].

Mena et al. [89] investigated Uros pharmacokinetics using an in vitro digestion model that involved oral, gastric, and pancreatic digestion, followed by 24 h fecal fermentation. The results showed that the stability of Uro-B was much higher compared to Uro-B-gluc and Uro-A. The protein transporter plays a significant role in determining the biological distribution of Uro-B [92].

Techniques that guarantee the most efficient extraction of biological materials, whether they are fluids or tissues, are necessary for achieving the most precise evaluation of Uros from biological samples. In order to achieve this objective, the selection of appropriate extraction protocols that consider the unique properties of various matrices, as well as the presence of conjugated and non-conjugated forms of Uros, is necessary. To facilitate protein precipitation, plasma [25,31,32,33,34,36,37,38,42,44], breast milk [38], bile [44], and tissue samples, including brain [51] and prostate [31] tissue, were extracted using solvents such as MeOH and ACN (acidified with formic acid). Before analysis, urine samples were extracted using methanol [25], diethyl ether [32], or dilution with only water containing 0.1% formic acid [33,34,37,45]. In vitro fecal fermentation cultures were extracted with diethyl ether [25] and fecal samples were extracted with MeOH [44], employing ESI sources to detect and quantify analytes [34].

Furthermore, after extraction, Uros need to be identified and quantified. The most frequently employed methods for the separation of Uros were HPLC and UPLC, which used reversed-phase columns with octadecyl-bonded stationary phases and isocratic or gradient mobile phases that consisted of water mixed with acetonitrile or methanol with the addition of formic or acetic acid. UV diode array detectors (DADs) [25,31,33,34,38] were usually employed in tandem with mass spectrometers, including triple quadrupoles (QqQ) [34,42,45,46,49] and quadrupole–time of flight (QTOF) [34,36,37,38] systems.

#### 3.2.5. Metabolic Compounds Derived from Walnuts 

The metabolic compounds that were identified and/or quantified after walnut consumption [24,25,31,32,33,34,36,37,38,42,44,45,46,49,51] are represented in Table 3.

The compounds derived from the metabolism of ETs and EA present in walnut extracts were examined, both in vitro experiments using fecal microflora samples and in vivo murinic investigations. Additionally, biological samples obtained from healthy individuals and patients with different conditions were examined in several clinical studies (Table 3).

The main metabolite of EA is Uro-A (3,8-dihydroxy-6H-dibenzo[b,d]pyran-6-one), which is most frequently identified and quantified in humans in both blood and urine after consuming dietary ETs from walnuts (Figure 2) [32,34,42,46,49].

Cerda et al. [25] examined fecal samples from six healthy individuals to assess the ability of the human fecal microbiota to metabolize the EA punicalagin, which is a prevalent ellagitannin found in pomegranate (*Punica granatum* L.) [10] as well as an ET-concentrated extract from walnuts. A previous analysis of the extract’s phytochemical composition revealed the presence of several ETs, which are primarily located in the walnut skin [24]. All fecal cultures produced Uro-A during the metabolization process; however, the rates and concentrations of production differed greatly between the cultures. Nonetheless, there were notable differences in the amounts and rates of Uro-A production between the cultures. The composition of GM has a significant impact on the synthesis of Uros, which can vary considerably among individuals [25].

The defatted walnut powder extract (DWPE) is rich in many bioactive components, including phenolic acids like GA, EA, and isostrictinin [11], and dicarboxylic acid glycosides (glansreginins A, B, and C) [39]. DWPE demonstrated large beneficial impacts against obesity, cholesterol gallstones, type 2 diabetes (T2D), and non-alcoholic fatty liver disease (NAFLD) due to its highly effective hypolipidemic, anti-inflammatory, and hypoglycemic properties [44]. Ren et al. [44] evaluated the DWPE metabolites in male SD rats using the UPLC-Q-Exactive Orbitrap MS method and evaluated the effectiveness of the compounds. In addition, they examined the potential effects of DWPE on NAFLD by developing a network connecting metabolites and diseases and assessing the associated pathways. It was found that GA, EA, and Gla A were the main metabolites of the 52 DWPE metabolites identified. It was observed that, in nitrogen metabolism, the DWPE may lower ammonia concentrations and raise the expressions of carbonic anhydrase 2 (CA2) and carbamoylphosphate synthetase (CPS1).

Alzheimer’s disease (AD) is the most common type of age-related disorder, with a high incidence (60–80%). It affects 5.3% of adults 65–74 years old, 13.1% of people 75–84 years old, and 33.3% of people 85 years of age and above [93]. An in vivo study investigated the therapeutic effects of walnuts active fractions, namely walnut kernel (WK), defatted walnut powder (DWP), walnut kernel oil (WO), walnut kernel protein (WKP), walnut kernel organic acid (WKOA), and walnut kernel polysaccharide (WKPS) extracts in the treatment of scopolamine-induced AD in ICR mice. In this research, UPLC-Q-Exactive Orbitrap MS was used to assess eight active components identified in the brain tissue of AD mice. Additionally, given their effectiveness, specificity, and measurability, Gla A, EA, and EA 4-*O* xyloside were thought to be effective for use as marker molecules of WK in conjunction with molecular docking investigations [51].

Several clinical trials examined the metabolic compounds produced as a result of walnut consumption, both in healthy individuals and in patients with different conditions. Various Uros were identified in biological materials, such as blood (plasma), urine, or feces samples, as well as breast milk or prostate tissues. Uro-A was the most commonly detected urolithin [32,34,38,42,46,49], along with Uro-B [32,34,91], and their conjugated forms made with glucuronic acid [31,34,91] and sulfate [91]]. Additionally, isoUro-A [32,34,91], Uro-C [34,49] and their conjugates [31], as well as Uro-D, Uro-M6, and Uro-M7 were detected [34].

In their research, Cerda et al. [24] examined the metabolic processes of several dietary ETs and EA derivatives in healthy human subjects. A total of 40 individuals, divided into four groups, ingested either strawberries, red raspberries, walnut kernels (35 g, single dose), or oak-aged red wine, which are all food items containing ETs. Following this consumption, five samples of urine were obtained at certain time intervals between 8 and 56 h. All participants showed the presence of the microbial metabolite Uro-B, conjugated with glucuronic acid, in fractions F3–F5. Regarding the consumed ETs, the mean percentage of metabolite excretion varied from 2.8% in strawberries to 16.6% in walnut extracts.

Gonzales-Sarrias et al. [31] conducted a randomized controlled trial (RCT) to determine if ETs or their metabolites were present in the human prostate after consuming 35 g of peeled walnut kernel (PWK) per day for three days. A total of 63 patients, diagnosed with either benign prostatic hyperplasia or prostate cancer (PCa), were divided into three groups: a control group and two experimental groups. Uro-A gluc was the primary metabolite found (up to 2 ng/g), along with traces of Uro-B gluc and dimethyl ellagic acid (DMEA), regardless of the source of the ETs, demonstrating that certain urolithin conjugates (glucuronides) and DMEA can reach and penetrate the human prostate gland when ET-rich food sources are consumed (Table 3) [31].

A randomized, crossover controlled-feeding trial was designed to investigate the time-course effects of a walnut-rich test meal on plasma OS, antioxidant activity, the concentration of α- and γ-tocopherols and total polyphenols, and the urinary excretion of phenolic metabolites. The walnut test meal (90 g WK, single dose) was compared to a meal made of refined ingredients without ETs. After following a phenolic diet for one day and fasting overnight, blood samples were taken before the test meals and at various intervals up to 24 h after ingestion. These samples were then analyzed for the urinary excretion of phenylacetate metabolites and Uro-A, and the walnut meal led to a substantial increase in the excretion of Uro-A in urine (Table 3) [32].

Garcia-Villalba et al. [34] analyzed the UV and MS spectra characteristics of Uros, and their phase II metabolites were determined using various systems based on liquid chromatography (LC) coupled with diode array or mass spectrometer detectors employing different analyzers, as well as via QTOF and triple quadrupole (QqQ) techniques. Relative response factors (RRFs), characteristic UV spectra, and the elution order were determined in relation to EA and the most prevalent metabolite, Uro-A. The validated methods were effectively utilized on urine and feces samples via a human trial in which participants consumed foods containing ETs, such as walnuts (30 g PWK per day, 3 days) and pomegranate extracts. Following the consumption of walnuts, the levels of three Uros (Uro-A, Iso-A, and Uro-B) and their glucuronides were quantified in urine, while seven Uros were identified in fecal samples (Table 3) [34].

A comprehensive investigation was conducted to analyze the transmission of several Uros from mothers to their infants through breast milk, as well as the colonization of infants’ guts by the Uro-producing bacterium *Gordonibacter* throughout the first year of their lives. Walnuts (30 g PWK per day, 3 days) were used as a dietary source of urolithin precursors in two different studies: a proof-of-concept study with 11 participants and a validation study with 30 participants. Both studies were conducted on mothers who were breastfeeding their children. The total range of Uros in mothers’ breastmilk ranged from 8.5 to 176.9 nM. It was found that the mothers’ UM levels were responsible for determining the urolithin profile in breast milk, which can have biological implications for newborns [38].

In the DIRECT-PLUS study, 294 participants were randomly divided into three groups, each receiving a certain type of diet: one based on healthy dietary guidelines, the Mediterranean (MED) diet, or the green-MED diet. Each diet was combined with physical activity (PA). Both MED groups ingested 28 g of walnuts per day, which is equivalent to ating 440 mg of polyphenols per day. The green-MED diet group used green tea (3 to 4 cups per day) and Mankai (*Wolffia globosa* strain, one hundred grams of frozen cubes each day) green shake (800 mg of polyphenols per day) [42,46,49]. In that extensive trial, Uro-A [46,49] as well as Uro-C [49] were identified in the urine samples of all participants from both MED diet groups. An analysis of urine polyphenol compounds revealed a strong correlation between increases in Uro-A and reductions in visceral adipose tissue (VAT), even after factoring in multiple comparisons for the 139 identified metabolites (r = −0.241, *p* < 0.001). There was a significant correlation between the increase in Uro-A and the consumption of walnuts (r = 0.14, *p* = 0.035) and Mankai (r = 0.24, *p* = 0.044) [46].

From the perspective of categorizing different metabotypes in the participants, all the clinical studies used the same quantity and rate of walnut administration: 30–33 g over a three-day period. The final metabolic compounds were identified and quantified using HPLC-DAD-ESI-Q-MS or UPLC-ESI-QTOF-MS analytical methods. All the clinical studies, except for one [45], involved healthy volunteers. The percentages of metabotypes in the two administered categories of walnuts, PWK and WK [37,38], showed considerable similarity in the results. For instance, in PWK, UM-A was detected in 44% to 52% of the participants, UM-B was found in 43% to 55% of the participants, and UM-0 was not detected in any participant [37,38]. However, the experiments conducted with WK identified the percentages of UM-A between 65 and 70%, UM-B at 20%, and UM-0 which ranged from 10 to 15% [33,36]. The exception to those results was the study conducted by Romo-Vaquero et al. on PD patients, which revealed a reduced UM-A percentage of 45% [45].

### 3.3. Antioxidant Activity of ETs and Their Metabolites

OS was initially recognized as an imbalance between the antioxidants that protect the cell and the oxidants that assault it [94]. In a living cell or organism, OS generates an excessive amount of pro-oxidative species (primarily ROS and RNS) in comparison to antioxidant defenses (enzymatic and non-enzymatic) [95]. This imbalance results in the destruction of the main cellular macromolecules (carbohydrates, lipids, proteins, and DNA) to a varying degree, which in turn causes a gradual decline in the function of the organs and tissues [94]. It has been demonstrated that OS is a significant factor in the development of numerous chronic diseases, including cardiovascular, metabolic, and neurodegenerative disorders, and cancer, as well as in the aging process [95]. The sources of reactive oxygen and nitrogen species can be endogenous (nicotinamide adenine dinucleotide phosphate (NADPH) oxidase, myeloperoxidase (MPO), lipoxygenase, and angiotensin II) and exogenous (air and water pollution, tobacco, alcohol, heavy or transition metals, and some drugs) [95]. Antioxidant defense safeguards biological systems from free radical toxicity and encompasses both endogenous (enzymatic and nonenzymatic) and exogenous molecules. SOD, CAT, and glutathione peroxidase are the primary antioxidant enzymes [96], while bilirubin, α-tocopherol, and phenolic antioxidants, which include stilbene derivatives (as resveratrol), phenolic acids, and flavonoids, are exogenous antioxidants [97]. Plant-derived phenolic compounds are the primary natural antioxidant constituents present in food [98]. They serve as singlet oxygen suppressors, hydrogen atom suppliers, and free radical scavengers. Moreover, phenolic compounds can modulate several cell signaling pathways with important implications in antioxidant defense, including the activation of the nuclear factor erythroid 2/electrophile-responsive element (Nrf2/EpRE) pathway [99].

Walnuts are the seventh most significant source of total polyphenols among commonly consumed foods and beverages, as determined by the serving size [100,101]. In an investigation performed by Vinson and Cai [101], walnut polyphenols (WPhs) demonstrated the highest lipoprotein-related antioxidant activity of any of the examined constituents of nuts. The edible part of walnuts contains a large spectrum of phenolic compounds, such as phenolic acids, flavonoids, tannins, phenolic lignans, and stilbene derivatives, as evidenced by the phenolic profiles and antioxidant activities of free, esterified, and bound phenolics [41]. The ETs found in walnut, including pedunculagin, casuarictin, and Gla A, demonstrated a wide range of antioxidant properties [100]. Thus, a former in vitro gastric digestion simulation study showed that casuarictin and agrimoniin exerted anti-inflammatory activities via the inhibition of NF-κB pathway and IL-8 secretion, respectively [102]. Similarly, in a murinic model, ellagitannins including lambertianin and sanguiin efficiently protected against gastric ulcers via suppressing the pro-inflammatory cytokines and ROS [103]. More recently, in a model mimicking the gastric epithelium–*Helicobacter pylori* interaction, it was demonstrated that ellagitannin isomers, such as castalagin and vescalagin, presented anti-inflammatory activity through the attenuation of NF-κB signaling [104]. Upon reaching the duodenum, ETs are partially degraded into EA [105].

However, due to their poor absorption, EA and residual ETs may also penetrate the remaining portion of the small intestine [106]. The GM in the lower GI tract metabolizes EA and residual ETs to generate Uros, which, combined with their conjugate derivates, remain at relatively high concentrations in plasma and urine for days following the ingestion of dietary ETs [105]. Consequently, ETs and EA may provide local health benefits to the GI tract, while the systemic health benefits are more likely to be conferred by Uros [105].

Walnuts are known to have a variety of health benefits, including antioxidant, anti-inflammatory, anti-atherogenic, and anti-aging properties, as a result of their high content in terms of polyunsaturated fatty acids (linoleic and linolenic acids), as well as tocopherols and polyphenols, including EA and ETs [99]. Preclinical studies are an essential source of evidence for demonstrating these effects, elucidating the mechanisms of action, establishing dose–response relationships, and identifying the bioactive components responsible for beneficial effects. The primary findings of a selection of studies from the literature, focusing on the involvement of ETs and EA in walnuts, either as pure chemicals or in the form of extracts rich in ETs that have been isolated from walnuts and characterized phytochemically in various experimental models in vitro or in vivo, are presented below. The purpose of this summary is to describe the health benefits of walnuts and to identify their specific mechanisms of action.

The most prevalent Uros that can be detected or quantified in humans and animals are Uro-A, isoUro-A, and Uro-B. They have been identified as potent antioxidants through the oxygen radical absorbance capacity (ORAC) in vitro assay [19]. Uros demonstrated uncertain antioxidant activity in other assays, including 2,2-diphenyl-1-picrylhydrazyl (DPPH), the ferric reducing ability of plasma (FRAP), and 2,2′-azinobis-(3-ethylbenzothiazoline-6-sulfonic acid) (ABTS^+^) assays [107]. In this regard, Uro-A, which was tested as a direct radical scavenger, exhibited lower activity than EA [19]. But the DPPH, FRAP, and ABTS assays are based on a different mechanism than the ORAC test. Thus, whereas the first three tests are based on the same mechanism, i.e., single-electron transfer, and thus assess antioxidant activity by scavenging a radical (DPPH, ABTS) or by the reduction of metal ions (FRAP), the ORAC test measures antioxidant activity by the inhibition of peroxyl radical-induced oxidation, thus reflecting the classical antioxidant activity of radical chain breaking by hydrogen atom transfer [10].

The purpose of the study conducted by Haddad et al. [32] was to investigate the acute effects of ingesting walnuts in comparison to refined fat on OS that is generated by meals and to elucidate whether bioavailable ETs and tocopherols from walnuts offer postprandial antioxidant protection. In this randomized, crossover, and controlled-feeding study, the blood samples were collected prior to the test meals and at intervals of up to 24 h after ingestion, following the consumption of a low-phenol diet for one day and an overnight fast. The samples were analyzed for total phenols, malondialdehyde (MDA), oxidized LDL, FRAP, hydrophilic and lipophilic ORAC, uric acid, catechins, and the urinary excretion of phenylacetate metabolites and Uro-A. After the walnut test meal, the incremental area under the curve (AUC0–5 h) for hydrophilic ORAC and lipophilic ORAC increased by 7.5% and 8.5%, respectively, and was reduced by 7.4% for MDA. The plasma concentrations of gallocatechin gallate, epicatechin gallate, and epigallocatechin gallate increased significantly one hour after the walnut test meal. The walnut meal resulted in a significant increase in the quantity of Uro-A excreted in the urine [32].

**Figure 3:**
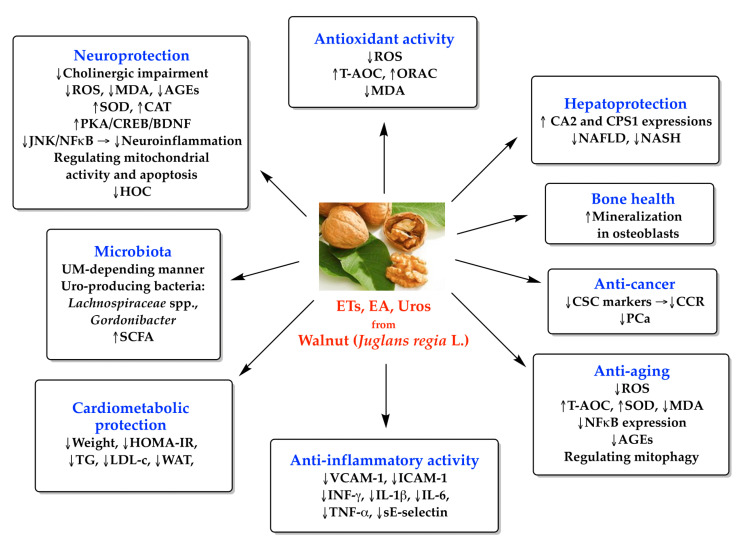
The mechanisms of ellagitannins, ellagic acid, and urolithins and their multiple beneficial health effects after walnut consumption (AGEs—advanced glycation end products; BDNF—brain-derived neurotrophic factor; CA2—carbonic anhydrase 2; CAT—catalase; CRC—colorectal cancer; CPS1—carbamoylphosphate synthetase; CREB—cAMP-response element binding protein; CSCs—cancer stem cells; EA—ellagic acid; ETs—ellagitannins; HOC—hippocampal occupancy score; HOMA-IR—Homeostatic Model Assessment for Insulin Resistance; ICAM-1—intracellular adhesion molecule 1; IL—interleukin; INF-γ—interferon gamma; LDL-c—low-density lipoprotein cholesterol; MDA—malondialdehyde; NAFLD—non-alcoholic fatty liver disease; NF-κB—nuclear factor kappa-light-chain-enhancer of activated B cells; NASH—non-alcoholic steatohepatitis; ORAC—oxygen radical absorbance capacity; PCa—prostate cancer; PKA—protein kinase A; ROS—reactive oxygen species; SCFA—short-chain fatty acids; SOD—superoxide dismutase; TG—triglycerides; TNFα—tumor necrosis factor-α; T-AOC—total antioxidant capacity; UM—urolithin metabotypes; Uros—urolithins; VCAM-1—vascular cell adhesion molecule 1; WAT—white adipose tissue; ↑—increases; ↓—decreases).

Many of the other biologically beneficial health effects of polyphenols, including ETs, EA, and Uros, occur based on the antioxidant action exerted by various mechanisms (the main ones are mentioned above). Often, preclinical and clinical studies investigate complex pathways of action, and the results obtained reflect, at least in part, the correlation between antioxidant and anti-inflammatory, neuroprotective or cardioprotective, anti-cancer, anti-aging, and antimicrobial action (Figure 3). In the following section, we point out these correlations, because they are of particular importance in understanding the mechanisms of action and the biological effects of walnuts and their bioactive compounds.

### 3.4. Anti-Inflammatory Activity of ETs and Their Metabolites

The biological process of inflammation has evolved to enable organisms to detect and respond to both beneficial and excessive stresses induced by internal or external stimuli [108]. The regulation of this process is governed by intricate immunological, neurological, and hormonal processes [109]. As a result of disruptions in these mechanisms, a diverse array of acute and chronic inflammatory conditions, including infectious diseases, critical illnesses, cardiovascular disease (CVD), cancer, and autoimmune diseases, collectively have a significant global health impact [110]. The inflammatory cytokines, including tumor necrosis factor-α (TNF-α), interferon-γ (IFN-γ), interleukin-1β (IL-1β), and IL-6, are released by a diverse array of cell types, particularly macrophages and mast cells. Their involvement in the inflammatory response is complex, including the activation of the endothelium and leukocytes, as well as the initiation of the acute-phase response [108].

Inflammatory processes can be initiated within cells by a variety of factors, which are stimulated by OS. This can result in the direct release of inflammatory mediators into the bloodstream, or the indirect activation of genes and signals associated with inflammatory conditions [108]. In fact, OS can activate a variety of transcription factors, such as p53, nuclear factor kappa-light-chain-enhancer of activated B cells (NF-κB), PPAR-γ, activator protein-1 (AP-1), and hypoxia-inducible factor-1α (HIF-1α). The activation of these factors has been demonstrated to induce the expression of a variety of genes that encode cytokines, chemokines, cell cycle regulatory molecules, growth factors, and anti-inflammatory molecules [108].

Numerous research investigations have shown the potential of plant-based diets and foods, which include walnuts, to lower the risk of developing non-communicable diseases and comorbidities, as well as their incidence and mortality [111]. Scientific evidence has shown that walnuts contain a vast assortment of phytochemical compounds and micronutrients that provide protection against inflammation, including ω-3 and ω-6 polyunsaturated fatty acids, tocopherols, antioxidant polyphenols (catechins, resveratrol, ETs), phytosterols (stigmasterol, campesterol, sitosterol), and active prebiotics [74,100,112].

#### 3.4.1. Preclinical Studies

The cellular accumulation of ROS activates the NF-κB transcription factor pathway, which in turn controls the expression of numerous inflammation mediators. Hence, OS and the consequent inflammatory response are the fundamental causes of the occurrence or worsening of degenerative pathologies, allergic reactions, and autoimmune disorders. This explains why diets that are high in antioxidants, such as the MED diet, are negatively correlated with pathological conditions and positively correlated with lifespan and healthspan. Walnuts, which are essential components of the MED diet, are known to modulate the inflammatory response and alleviate or combat the inflammation–aging state, namely, “inflamm-aging”, which is characterized by chronic, low-grade inflammation and is associated with aging [5,99].

Inflammatory and atherogenic processes are initiated during the early stages of CVD development, while the adhesion molecules are activated by cytokines. Papoutsi et al. [29] examined the impact of EA, a critical component of a walnut methanolic extract, on the expression of the vascular cell adhesion molecule, VCAM-1, and the intracellular adhesion molecular collection, ICAM-1. The assay was conducted in vitro on human aorta endothelial cells (HAECs) that had been activated by exposure to TNF-α. The expression of both VCAM-1 and ICAM-1 was reduced by the methanolic walnut extract (10–200 μg 4-methyl-catechol equivalents/mL) compared to the control (*p* < 0.001). EA exhibited a comparable, efficacious effect on VCAM-1 (*p* < 0.01 vs. control) over an increased concentration range (0.1–10 μM) compared to its effect on ICAM-1 (*p* < 0.05 vs. control, at 0.1–1 µM). Consequently, Papoutsi et al. [29] illustrated the substantial anti-atherogenic potential of walnuts and the potential contribution, at least in part, to this effect by the EA present in walnuts. Therefore, this research demonstrated that walnut consumption has a positive impact on cardioprotection and the preservation of endothelial function [29].

In another study, Anderson et al. [30] investigated the effects of two walnut polyphenol extracts (WPhEs), one that was cold-obtained (CE) and another that was hot-prepared (HE), in parallel with EA on the proliferation of peripheral blood mononuclear cells in human peripheral blood (PBMCs) that were activated by a variety of stimulants. Both extracts, as well as EA, significantly suppressed in vitro human PBMC proliferation that was stimulated by phytohemaglutinine (PHA), α-CD3, or phorbol miristat acetate (PMA)/ionomycin in a dose-dependent manner. There was variation in the modulation of cytokine production by PBMCs that were stimulated by PHA. When it came to TNF-α levels, both WPhEs (CE and HE) reduced TNF-α levels, but the hot-prepared extract was 10 times more active than the cold-obtained one (*p* ≤ 0.05 for 100 μM GAE CE and 10 μM GAE HE, respectively). The levels of IL-13 were decreased by both WPhEs and EA to a similar extent (*p* ≤ 0.05 for 100 μM GAE CE or HE and 100 μM EA); however, there was no effect on IL-4. The regulation of the two ILs is distinct, and it seems that the polyphenols in walnut only disrupt the regulatory mechanism of IL-13, not IL-4. However, EA (100 μM) was found to enhance IL-2 levels (*p* ≤ 0.05), while WPhEs did not have the same effect [30]. Thus, the immunomodulatory capacity and anti-inflammatory potential of polyphenols in walnuts are dependent on the degree of immune cell activation, the conditions of immunostimulation in the environment, and the dose used.

#### 3.4.2. Clinical Studies

Research has demonstrated that walnut supplementation, when associated with other foods and nutrients, can reduce the overall adverse effects of inflammation [74]. The WAHA (Walnuts and Healthy Aging) study was a randomized clinical trial that employed systemic biomarkers to determine whether the consumption of walnuts reduced inflammation in elderly individuals [113]. Over a two-year period, participants adhered to a rich walnut diet (15% energy; 30–60 g daily). In comparison to the control group, there was a significant decrease in the levels of several inflammatory markers, including IFN-γ and IL-1β (*p* < 0.001), IL-6, TNF-α, and sE-selectin (*p* < 0.05) [113]. In addition, a recent meta-analysis of randomized controlled studies reported the effects of consuming walnuts on several metabolic syndrome and inflammatory markers in middle-aged and older individuals [114]. Consequently, walnut consumption resulted in significant changes in several inflammatory biomarkers, including IFN-γ, IL-6, L-1β, and E-selectin (*p* < 0.001), along with TNF-α (*p* = 0.009). However, no significant influence on C-reactive protein (CRP) or high-sensitivity CRP (hs-CRP) was observed [114].

Although urolithin aglycone is highly bioactive against inflammation, some authors propose that the urolithin conjugate may be even more effective [78]. In an investigation performed by Piwowarski et al. [115], a volunteer consumed pomegranate juice (0.5 L/day), walnuts (30 g/day), hazelnuts (30 g/day), and fresh raspberries (200 g/day) for 5 days. The cleavage of glucuronides by endogenous β-glucuronidase (an enzyme that is present at high concentrations in the microenvironments of solid tumors and at inflammation and infection sites) was observed. Urolithin conjugates (iso-Uro-A-gluc, Uro-A-gluc, and Uro-B-gluc) were isolated from the volunteer’s urine. The hypothesis that the selective activation of urolithin glucuronides by β-glucuronidase could locally increase the concentration of bioactive urolithin aglycones is supported by these results. However, to gain a more comprehensive understanding of the anti-inflammatory response associated with urolithin and its impact on the suppression of immune responses, additional clinical trials are required [116].

### 3.5. Cardiometabolic Activity of ETs and Their Metabolites

Metabolic syndrome (MetS), also known as Syndrome X and the Insulin Resistance Syndrome, is a widespread health issue that encompasses various cardiovascular conditions such as heart attack, stroke, and atherosclerosis, as well as endocrine disorders like obesity, NAFLD, insulin resistance, and diabetes [117]. According to the National Cholesterol Education Program-Adult Treatment Panel III and the International Diabetes Federation protocols [118], a positive diagnosis for MetS requires the presence of three or more factors, along with central obesity (waist circumference ≥90 cm for males or ≥85 cm for females). They include: (1) hypertriglyceridemia (serum triglyceride concentration ≥ 150 mg/dL); (2) low levels of high-density lipoprotein cholesterol (HDL-c) (<40 mg/dL for men; <50 mg/dL for women); (3) high blood pressure, ≥130/85 mmHg; and (4) elevated fasting blood glucose levels (>100 mg/dL) [117].

MetS has a multifaceted etiology that encompasses other factors, including OS, systemic inflammation, and GM dysbiosis. These characteristics are particularly significant because they have the potential to produce systemic effects that interact with conventional MetS components, such as insulin resistance or blood pressure [119]. Cardiometabolic risk (CMR) is a term that refers to clinical abnormalities, including hyperinsulinemia, abdominal adiposity, atherogenic dyslipidemia, and elevated blood pressure, that are indicative of chronic diseases, including CVD and/or T2D [120].

The association between UMs and CMR factors was investigated in individuals with varying body mass index (BMI) and health status [36]. In this research, the associations between basal CMR factors and the urine urolithin metabolomic signature were evaluated for 20 healthy normoweight individuals who consumed 30 g of walnuts per day, 49 healthy overweight–obese individuals who consumed 45 mg of pomegranate extract per day, and 25 MetS patients who consumed nuts (15 g of walnuts, 7.5 g of hazelnuts, and 7.5 g of almonds per day). The study identified correlations between Uros and CMR factors in overweight–obese individuals. Uro-A, which is predominantly present in UM-A, demonstrated a positive correlation with apolipoprotein A-I (*p* ≤ 0.05) and intermediate-HDL-c (*p* ≤ 0.05). Conversely, Uro-B and isoUro-A, which are characteristic of UM-B, showed a positive correlation with triglycerides (TG), low-density lipoprotein cholesterol (LDL-c) (*p* ≤ 0.001), apolipoprotein B (*p* ≤ 0.01), very-low-density lipoprotein cholesterol (VLDL-c), intermediate-density lipoprotein cholesterol (IDL-c), oxidized LDL-c, and the apolipoprotein B/apolipoprotein A-I ratio (*p* ≤ 0.05). The risk of CMD was elevated in overweight–obese individuals with UM-B, while the production of Uro-A could provide protection against CMR factors [36].

#### 3.5.1. Weight, Waist Circumference, Visceral Adiposity

Obesity and CMD are becoming more widely recognized as conditions that are significantly influenced by the GM [121]. CMD includes several conditions, such as high blood pressure, atherosclerosis, heart failure, obesity, and T2D [122]. New research on the connection between CMD and GM has expanded our knowledge of how diet and nutrition impact microorganisms, which in turn affect CMD [123]. Uros have been recognized as possible contributing factors to the beneficial effects against CVD associated with the consumption of ET-containing foods [124].

Zelicha et al. [46] investigated the influence of the green-MED diet, which is additionally high in dietary polyphenols and contains a lower amount of red or processed meat, on visceral adipose tissue (VAT) along with the MED diet. Overall, 294 participants were randomly assigned to one of three diets—(1) healthy dietary guidelines, (2) MED diet, or (3) green-MED diet. All of these were accompanied by physical activity and assessed in the 18-month Dietary Intervention Randomized Controlled Trial Polyphenols Unprocessed (DIRECT PLUS) weight-loss trial. MED diet groups that were both isocaloric consumed 28 g of walnuts per day (440 mg of polyphenols per day). The green-MED diet group consumed green tea (3–4 cups daily) and a drink made from the *Wolffia globosa* (800 mg of polyphenols per day), and they reduced their consumption of red meat. The amount of abdominal fat tissue was measured using magnetic resonance imaging (MRI). VAT loss was statistically significant (*p* < 0.05), independent of age, sex, waist circumference, or weight loss. Therefore, the consumption of the green-MED diet and the increased levels of urine Uro-A were found to be significantly correlated with a greater loss of VAT (*p* < 0.05, in multivariate models) [18].

#### 3.5.2. Gut Health

There are specific distinctions between the terms “microbiota” and “microbiome”, despite their frequent interchangeability. Microbiota refers to the living microorganisms that are present in a specific environment, such as the oral and intestinal microbiota [125]. Microbiome is the collective term for the genomes of all the microorganisms in the environment, encompassing not only the microbial population but also the structural components of microbes, metabolites, and environmental factors [88,126,127]. 

Among its numerous physiological functions, the participation of the GI microbiota in the food digestion process is particularly noteworthy. For instance, GM metabolize complex polysaccharides after they are initially digested by intestinal enzymes in the small intestine [128]. Undigested carbohydrates (CHO) and proteins in the intestinal lumen stimulate the metabolism of anaerobic bacteria, producing short-chain fatty acids (SCFAs) and gases such as hydrogen and methane [87]. SCFAs (butyrate, acetate, and propionate) act as a source of energy for intestinal cells, lower inflammatory responses, reduce the risk of developing colorectal cancer (CRC), and restrict the proliferation of pathogenic bacteria [129]. Acetate and butyrate activate GPR43 and GPR41 receptors on intestinal cells, thereby promoting the secretion of insulin, GLP-1, and peptide YY. This process assists in the regulation of blood lipid metabolism and reduces peripheral blood glucose levels [130].

GM can produce a variety of metabolites from a variety of dietary compounds. These metabolites have biological activity and are crucial for the maintenance of gut and metabolic health [131]. In the case of ETs and EA from pomegranate, walnuts, and berries, the human GM extensively metabolizes these compounds, as already shown, to generate various Uros with potential health benefits [18].

García-Mantrana et al. [37] investigated how a short (three-day) dietary intervention using walnuts affected the makeup and function of the GM in healthy participants based on their unique microbial profiles. The aim of the study was to determine whether short-term walnut consumption may be associated with differential gene modulation (GnM) in UMs. In this investigation, 27 healthy individuals consumed 33 g of peeled raw walnuts over the course of three days. GnM profiling was determined through 16S rRNA illuminasequencing and specific real-time quantitative polymerase chain reactions (qPCRs), and microbial activity was assessed using the short-chain fatty acid analysis of stool samples. It was observed that GM in UM-B’s population were more susceptible to walnut intervention. Some members of the *Lachnospiraceae* family decreased in UM-A individuals, while *Blautia*, *Bifidobacterium*, and members of the *Coriobacteriaceae* family, including *Gordonibacter*, increased exclusively in UM-B subjects (*p* < 0.05). The consumption of walnuts led to an increase in the production of acetate and propionate, as well as an increase in *Coprococcus* and *Collinsella* in both UMs [37]. The study revealed that the consumption of walnuts after only three days influences GnM in an UM-dependent manner and increases the production of SCFAs.

A separate study examined the maternal–infant transmission of multiple Uros through breast milk and the gut colonization of neonates by the urolithin-producing bacterium *Gordonibacter* during the first year of life [38]. Two trials were conducted, in which breastfeeding mothers ingested walnuts as a dietary source of urolithin precursors (proof-of-concept study: *n* = 11; validation study: *n* = 30). The urolithin profile in breast milk was influenced by the mothers’ UMs, which may have biological implications for neonates. *Gordonibacter* colonization of the gut of neonates during the first year of life was monitored via a qPCR method [38].

### 3.6. Neuroprotection Activity of ETs and Their Metabolites

Neuroprotection is mediated by antioxidant activity at the cellular level through a variety of biological mechanisms. When these mechanisms are elucidated, they can be utilized as therapeutic targets. Neuroinflammation is an innate immunological response of the central nervous system (CNS) that occurs as an early event in neurodegenerative diseases [132]. Systemic inflammation may predispose the microglia and astrocytes to a pro-inflammatory state, which is associated with neurodegenerative diseases, including AD and moderate cognitive impairment [133]. Neurodegenerative diseases are significantly influenced by the aging process. This results in a neuroinflammatory response, which in turn induces microglia and astrocytes to generate free radicals and secrete pro-inflammatory cytokines [133]. Decreased cognitive function may result from the release of pro-inflammatory cytokines by the neuroinflammatory cascade, and the brains of AD patients exhibit elevated levels of pro-inflammatory cytokines, including TNF-α, IL-1, and IL–6 [134].

#### 3.6.1. Preclinical Studies

An et al. [47] studied the ability of WPhs and Uro-A to protect the human neuroblastoma SH-SY5Y cells against damage caused by exposure to H_2_O_2_ via an in vitro model used for neurodegenerative disorders [135]. SH-SY5Y cell viability was completely protected by WPhE pretreatment (*p* < 0.01 at 75–150 μg/mL). Uro-A had also a protective action, but in a reversed “U”-shape manner, with a maximum effect at 10 μM (*p* < 0.01), demonstrating a hormesis-type action. Both WPhEs (50 and 100 μg/mL) and Uro-A (5 and 10 μM) demonstrated anti-apoptotic activity, reducing the number of apoptotic cells and normalizing the nuclear chromatin morphology. The cell protective action is based on the antioxidant activity of polyphenols from walnuts, but also that of their metabolites. This was established because both WPhE and Uro-A pretreatments maintained high levels of SOD and CAT activities (*p* < 0.05), protected against extracellular LDH leakage and intracellular Ca overload, and prevented an increase in ROS levels (*p* < 0.01). Exploring the mechanisms of action involved in neuroprotection, it was demonstrated that walnuts stimulate the protein kinase A/cAMP-response element, binding the protein/brain-derived neurotrophic factor (PKA/CREB/BDNF) signaling pathway through the ETs they contain and the ETs’ metabolites, the Uros. Thus, WPhEs (50 and 100 μg/mL) and Uro-A (5 and 10 μM) stimulated the cAMP-dependent PKA activity (*p* < 0.01) and increased the pCREB (Ser133) and BDNF expressions (*p* < 0.01 for all, except pCREB at 5 μM Uro-A with *p* < 0.05). Moreover, PKA inhibitor H89 pretreatment abolished the protection provided by WPhEs (100 μg/mL) and Uro-A (10 μM) (*p* < 0.01), which confirms that ETs from walnuts are responsible for the stimulatory action of PKA/CREB/BDNF signaling pathways [47]. 

OS, through the accumulation of ROS, accelerates the aging process and contributes to the development of age-related pathologies including neurodegeneration. The cAMP-dependent protein kinase A is involved in the phosphorylation of CREB, an important transcription factor in neurogenesis, neuroplasticity, neuronal survival, and brain defense against OS. It is involved in the regulation of antioxidant genes, in the induction of anti-apoptotic genes, in learning, and in memory processes, particularly by regulating the brain’s levels of BDNF. The activation of the PKA/CREB/BDNF signaling pathway stimulates neuroprotection and its upregulation is a strategy with which to maintain normal neuronal functions and prevent brain damage during aging or under stress conditions. Other studies have also shown that CREB is a target of nut diets [136,137] associated with antioxidant and neurotrophic effects [137]. Research by An et al. [47], however, demonstrates that walnut ETs and Uros are activators of this pathway. While ETs have relatively low bioaccessibility in vivo and EA has a low capacity to cross the blood–brain barrier. Uros reach active concentrations in the brain and are mainly responsible for the neuroprotective effects of walnut consumption. These findings open new perspectives on exploiting the favorable potential of Uro-A in maintaining and protecting brain functions.

Moon et al. [43] studied the neuroprotective effect of an extract obtained from walnut kernels of a Gimcheon 1ho cultivar (GC) rich in EA and from ETs such as pedunculagin/casuariin isomers, strictinin, tellimagrandin I, and ellagic acid-*O*-pentoside. The in vitro study was conducted on two cell lines: the authors used the neuronal PC12 cell line, which is derived from rat pheochromocytoma and used as an in vitro model to study the neuronal differentiation [138], and from hippocampal HT22 cell lines, which are used to study the implication of cholinergic neurons in cognitive functions [139]. WPhE-GC (20 μg/mL and 50 μg/mL) was found to increase the cellular viability of both cell lines that were affected by OS caused by exposure to H_2_O_2_ (200 μM) and increased the glucose concentration (50 μM) (*p* < 0.05) and decreased the ROS levels (*p* < 0.05) in both exposure models of cell lines. Thus, the tested extract demonstrated significant potential in terms of cellular neuroprotection [43]. The authors continued with a complex in vivo study to assess the efficacy of the same WPhE-GC in treating cognitive impairment in C57BL/6 mice in a high-fat-diet (HFD)-induced diabetes model. Both the tested doses, 20 mg/kg bw (GC20) and 50 mg/kg bw (GC50), significantly restored the HFD-altered behavior and neuronal functions, the ability in the Y-maze test (*p* < 0.05), the short-term working memory in a passive avoidance test (*p* < 0.01), the spatial learning acquisition in the Morris Water Maze (MWM) test (*p* < 0.05) vs. the HFD group. The extract improved the serum lipidic profile: the LDL-c level was significantly reduced (*p* < 0.01 vs. HFD group) by both doses, and LDH activity and TG levels were also diminished, but only at the higher dose (*p* < 0.05 vs. HFD group). The serum’s antioxidant activity (determined by FRAP test) was increased and AGE levels were reduced in the GC50 group (both *p* < 0.05 vs. HFD). The neuroprotective effects of WPhE-GC observed in vitro were confirmed in vivo by multiple mechanisms: the attenuation of cholinergic impairment (*p* < 0.05 for both GC20 and GC50 vs. HFD); a decrease in the OS in cerebral tissues via the regulation of mitochondrial activity (*p* < 0.01 for both GC20 and GC50 vs. HFD); brain damage architecture via the synergic regulation of the protein expression involved in neuronal apoptosis (*p* < 0.05 for all determined proteins for GC50); and a reduction in neuroinflammation via the JNK/NFκB signaling pathway (*p* < 0.05 for all determined proteins for GC50) (Table 1) [43]. Therefore, HFD disrupts carbohydrate homeostasis by generating the overproduction of ROS and AGEs and causes inflammation by activating the signaling pathways of NF-κB. In diabetes, the development of insulin resistance causes increased levels of pro-inflammatory cytokine expression and consequently increases the permeability of BHE, which also contributes to neuroinflammation. The activation of the JNK/Akt signaling pathway is associated with the production of amyloid peptides and synaptic dysfunction. According to the study of Moon et al. [43], GC walnuts have the ability to alleviate these processes, improve memory, and remedy cognitive dysfunctions by regulating synaptic function. EA and ETs are bioactive molecules that are largely responsible for the neuroprotective effectiveness of walnuts, contributing to their antioxidant and anti-inflammatory effects [43].

Xu et al. [51] used molecular docking to investigate the relationship between the metabolites of walnut kernel organic acids (WKOA), found in the brains of rats with scopolamine-induced AD, and pathophysiological pathways implicated in neurodegenerescence. The results showed that biologically active metabolites can target multiple signaling pathways, resulting in synergistic effects such as the inhibition of cholinesterases (GlaA and its metabolites), a decrease in OS, and neuroinflammation (EA and ellagic acid 4-*O* xyloside). Xu et al. [51] produced several active fractions from the WK, including WO, DWP, WKP, WKOA, and WKPS. They evaluated these fractions in vivo in scopolamine-induced AD male ICR mice after intragastric administration (equivalent dose of 15.6 g raw W/kg) for 8 weeks, using donepezil as a positive control. In MWM testing, WO, WKOA, and WKPS reduced the escape latency time (*p* < 0.05 vs. the model). The attention time was improved by using WK (*p* < 0.001), DWP, and WKOA (*p* < 0.05 vs. the model). The use of WKOA significantly enhanced spatial memory vs. other groups. Cholinergic transmission was reestablished: WKOA (*p* < 0.01) and WKP (*p* < 0.05) restored acetylcholine (ACh) levels in the hippocampus vs. the model, and WO (*p* < 0.01), WKOA, and WKP (*p* < 0.0001) restored ACh levels in cerebral cortex vs. the model. All WK fractions, but not WK, had the capacity to reduce the MDA levels in the hippocampus and cortex: WO (*p* < 0.001) > DWP, WKP (*p* < 0.01) > WKOA, and WKPS (*p* < 0.05) vs. the model in the hippocampus, and WO, DWP, and WKOA (*p* < 0.0001) > WKP and WKPS (*p* < 0.001) vs. the cortex model, respectively. These molecular changes were confirmed via histopathological analysis. In particular, WKP and WKOA attenuated the scopolamine-induced lesions in mice brains. WKOA significant diminished the NF-κB protein level in the hippocampus and cortex (*p* < 0.05), while OA only did so in the hippocampus (*p* < 0.05). Consequently, WKOA is the most WK active fraction, having real neuroprotective potential. It could protect the brain against OS and mitigate neuroinflammation, preventing AD development [51].

#### 3.6.2. Clinical Studies

The long-term consumption of walnuts has been demonstrated to enhance cognitive function and memory in humans. In a clinical trial that lasted 6.5 years, healthy adults that were aged 67–75 years and not affected by any cognitive impairment were given 15 g of walnuts daily as a supplement. In comparison to a control group that did not consume walnuts in their daily diet, walnut consumption resulted in improved cognitive function and memory [140]. The cognitive function of healthy adult subjects on a MED diet supplemented with 30 g mixed nuts/day (15 g walnuts, 7.5 g hazelnuts, and 7.5 g almonds) was superior to that of the control group on a low-fat diet in two clinical trials. Additionally, memory was significantly enhanced when compared to baseline scores in the control group [140].

In the RCT performed by Kaplan et al. [42], it was observed that a green-MED diet (a diet low in red or processed diet, rich in Mankai, green tea, and walnuts, and high in polyphenols) has neuroprotective potential for age-related brain atrophy. In this 18-month clinical trial, the brain structure volumes were longitudinally measured using MRI, with the hippocampal occupancy score (HOC) and lateral ventricle volume (LVV) expansion score serving as neurodegenerative markers. In the entire sample and in subjects aged 50 years or older, elevated concentrations of urine urolithin and tyrosol were significantly associated with a reduced decline in the HOC. During the trial, the parameter that was most strongly associated with the attenuation of brain atrophy was improved insulin sensitivity (*p* < 0.05). The consumption of the green-MED diet, including 28 g walnuts per day, was found to be significantly and independently associated with a decrease in HOC (*p* < 0.05). A slowing of the decline of HOC was strongly associated with increased levels of Uro-A (*p* = 0.013) and tyrosol (*p* = 0.007) in urine.

PD is a neurodegenerative condition that is distinguished by the loss of dopaminergic neurons in the midbrain, particularly in the substantia nigra, and by the presence of Lewy body inclusions in the brain [141].

Alterations to the GM (gut dysbiosis) are observed during the onset and progression of PD. Uros, anti-inflammatory metabolites that are synthesized from certain dietary polyphenols by specific gut microbial ecologies (urolithin metabolism types), have been suggested as biomarkers of the composition and functionality of the GM [45]. The research performed by Romo-Vaquero et al. [45] evaluated the connections between UMs, intestinal dysbiosis, and the severity of PD in individuals. Feces samples were collected from the participants (52 patients and 117 healthy controls) for microbiota sequencing and urine samples for urolithin profiling prior to and following the consumption of 30 g of walnuts over a three-day period. The GM of UM-0 patients (Table 3) and patients with the highest severity was distinguished by a more altered bacterial composition, which is defined as an increase in pro-inflammatory *Enterobacteriaceae* and a decrease in protective bacteria against autoimmune and inflammatory processes. These bacteria included butyrate and urolithin-producing bacteria (*Lachnospiraceae* and *Gordonibacter*) [45].

### 3.7. Antitumoral Potential of ETs and Their Metabolites

Cancer is a significant global public health hazard and a leading cause of mortality [142]. The recent evidence suggests that, by avoiding potential modifiable risk factors, such as tobacco use, alcohol consumption, an improper diet, a lack of exercise, and infectious agents, approximately 40% of cancer cases can be prevented [143]. It was demonstrated that adhering to a healthy diet, such as the MED diet, can decrease the risk of certain types of cancer by 4% to 57% [143].

Numerous studies have indicated that the consumption of tree nuts (walnuts, almonds, hazelnuts, cashews, pistachios, and pecans) may have a beneficial effect on long-term health by reducing the intermediate mediators of chronic diseases, including insulin resistance, inflammation, hyperglycemia, and OS [142]. Several nut compounds, including resveratrol, EA, anacardic acid, and ω-3 and ω-9 fatty acids, have been shown to induce cancer cell death, inhibit the proliferative capacity of cancer cells, and reduce metastasis by inhibiting critical cancer molecular pathways, including NF-κB, metalloproteinases, and the VEGF family. Therefore, nuts possess a broad-spectrum anticancer effect, even though the precise mechanisms behind this have not yet been completely elucidated [142].

#### 3.7.1. Preclinical Studies

Cancer stem cells (CSCs) are responsible for relapses and resistance to anti-tumor medication in CRC. They possess a high capacity for self-renewal and tumor regeneration. In their study, Lee et al. [35] examined the ability of a WPhE to inhibit CSCs. They also measured the levels of the main bioactive compounds in this extract, namely, (+)-catechin, chlorogenic acid, EA, and GA, which were present in concentrations comparable to 40 μg/mL of WPhE. The quantities of these compounds were found to be 137.5 mg/100 g, 13.6 mg/100 g, 12.6 mg/100 g, and 10.7 mg/100 g, respectively. Tests were performed on CD133+CD44+ cells, phenotypes with strong tumorigenic potential, that were obtained from the human CRC HCT116 cell line. WPhE inhibited the survival of CD133+CD44+HCT116 cells (*p* < 0.01 vs. control at 40 μg/mL, after 2, 4, and 6 days) and induced cellular differentiation by increasing cytokeratin 20 (CK20) expression by 164% (*p* < 0.0001 vs. control at 40 μg/mL, after 6 days). When compared to the control group, WPhE (40 μg/mL) significantly reduced the levels of several CSC markers, including CD133 and CD44; delta-like protein 1 (DLK1) in *Drosophila*, which functions similarly to epidermal growth factor (EGF) and is involved in cell differentiation; and the level of Notch1, a single transmembranar receptor involved in stem cell maintenance, cell proliferation, and apoptosis. The self-renewal capacity of CSC was inhibited by WPhE, which downregulated the (+)-catenine/p-GSK3 signaling pathway and inhibited colony formation and non-adherent spheroid formation by up to 94% and 72.3%, respectively (*p* < 0.001 vs. control). WPhE exhibited more potent anti-CSC effects (*p* < 0.05 for each effect examined), which varied according to dose when compared to the individual bioactive chemicals examined. In colon primary cancer cell cultures derived from primary colon cancers, the WPhE suppression of CSC markers was demonstrated (*p* < 0.05). Consequently, WPhE may inhibit the growth of CRC by controlling the properties of CSC in the colon, raising possibilities for the use of WPhs in CRC prevention and treatment strategies [35].

#### 3.7.2. Clinical Studies

PCa is the second most frequently detected form of cancer [144] and the most prevalent cancer among males in numerous industrialized countries, being associated with a significant mortality rate [145]. The only well-established risk factors for PCa are older age, black ethnicity, and a family history of the disease [146]. There is evidence that a high-calcium diet, dairy products, adult achieved height, low plasma selenium and alpha-tocopherol concentrations, and body fatness all raise the risk of PCa [147]. 

González-Sarrías et al. [31] investigated the effects of ETs, EA, Uros, or any other derived conjugate that can be detected and measured in the human prostate gland of PCa and benign prostate hypertrophy male patients after consuming pomegranate juice (200 mL per day) or walnuts (35 g per day) 3 days before surgery. Although both Uro-A gluc and Uro-B gluc were detected in the analyzed prostate tissues, there were no apparent changes in the expression of CDKN1A, MKi-67, or c-Myc following the consumption of the walnuts or pomegranate juice [31]. Uros exerted their chemopreventive effects on PCa in a dose-dependent way, which is linked to apoptosis induction, the upregulation of p21 expression, and cell cycle arrest [100].

### 3.8. Other Potential Therapeutic Effects of ETs and Their Metabolites

#### 3.8.1. Hepatoprotective Effects

Ren et al. [44] investigated the mechanisms that explain how a DWPE affects NAFLD in an in vivo animal model using HFD-induced C57BL/6 mice. They initially assessed the phytochemical profile of DWPE and identified extract metabolites in SD rats. The metabolites were used to perform network pharmacological screening in order to confirm the correlations between their chemical structures and various pharmacological pathways. They identified 11 potential pathological pathways connected to 54 biological targets. They started from 52 metabolites identified in vivo, the majority of which were active metabolites derived from GA, EA, and Gla A. These pathological pathways include those involved in the development of NAFLD, mainly lipid metabolism, signaling pathways involved in inflammation, and nitrogen metabolism. NAFLD is a prevalent pathology worldwide, with a prevalence of 32% in adults [148]. It is caused by OS, which disrupts lipid metabolism [149]. NAFLD may co-occur with non-alcoholic steatohepatitis (NASH), liver cirrhosis, or even liver cancer. Subsequently, the results of the high-grade in silico correlation were verified in vitro, in HepG2 cell cultures, and in vivo for C57BL/6 mice that were administered an HFD. DWPE (100 μg/mL) showed a hepatoprotective effect against OA-induced steatosis in HepG2 cells, reducing the buildup of lipids inside the cells (*p* < 0.001 vs. control) and the concentration of ammonia within the cells (*p* < 0.05 vs. control). This effect was similar to that of the positive control, L-ornitine L-aspartate (LOLA) (50 μM, *p* < 0.05 vs. control for both measurements) [44].

The CA2 and CPS1 expressions were significant increased by DWPE as well as by LOLA (*p* < 0.05 vs. control). CA2 is an important enzyme involved in maintaining acid–base homeostasis in the body and CPS1 is the first enzyme involved in urea cycle. In vivo DWPE (1.2 g/kg bw) administration in HFD-induced C57BL/6 mice improved the lipid profile, diminished TG and TC serum levels (*p* < 0.05 vs. HFD group), reduced serum ammonia concentrations (*p* < 0.01), and augmented the CA2 (*p* < 0.05) and CPS1 (*p* < 0.01) expression in liver, as seen with LOLA granules (*p* < 0.01 vs. HFD group). Consequently, DWPE has the capacity to reduce or prevent inflammation by intensifying nitrogen metabolism by converting excess nitrogen accumulated in cells into urea, alleviating OS at the liver level. The research demonstrates that DWPE has protective effects against NADFL and NASH and has the advantage of emphasizing the multiple mechanisms through which DWPE intervenes in the physiopathological pathways of these diseases [44].

#### 3.8.2. Bone Health

Papoutsi et al. [29] tested the same methanolic extract from walnut that exhibited anti-inflammatory activity in HAEC cultures activated with TNF-β, this time in KS483 osteoblastic cell cultures. This cellular line has the capacity to generate in vitro mineralizing nodules and facilitates the identification of bioactive compounds that promote the formation of osteoblasts. Osteoporosis is prevalent among elderly individuals, particularly women who have experienced menopause; however, it is less prevalent in the Mediterranean region. Polyphenols and polyunsaturated fatty acids, which are the primary components of walnuts, have beneficial effects on bone health. They participate in an array of bone function processes, which include stimulating bone differentiation and formation, thus reducing bone resorption and apoptosis. Papoutsi et al. [29] observed a substantial increase in the number of mineralizing nodules formed in osteoblasts in exposed KS483 cells in both walnut (10–25 μg/mL) and EA (1–10 nM) (both *p* > 0.05 vs. control) in their study. Accordingly, at concentrations where they have a protective effect against the vascular endothelium, the extracts of phenolic compounds from walnuts accelerate bone mineralization, with EA being a major factor in this process [28].

#### 3.8.3. Anti-Aging Effects

Cellular senescence and the accumulation of cellular dysfunction are the hallmarks of the aging process, which leads to the systemic deterioration of tissue. Increased fragility and the eventual development of age-related neurodegenerative, musculoskeletal, cardiovascular, integumentary, and neoplastic diseases are the consequences of this systemic loss of functional tissue [150].

In senescent tissues, a variety of structural and functional alterations within mitochondria are exhibited, such as disruptions of oxidative phosphorylation and the accumulation of mitochondrial deoxyribonucleic acid mutations [150]. The mitochondrial membrane potential is altered during senescence, resulting in the loss of the proton gradient, which can result in an imbalance between the supply and demand of energy as well as an increase in the production of ROS [150]. These elevated levels of ROS result in local tissue injury through inflammatory pathways and contribute to neuronal necrosis (observed in neurodegenerative diseases), and to the accumulation of abnormal mitochondria (specifically in sarcopenia) [151]. Increasingly, scientific data indicate that mitochondrial malfunction has a significant role in the aging process and the subsequent onset of age-related diseases [150].

##### Preclinical Studies

Tian et al. [40] studied the anti-aging potential of hydrolysates, obtained in vitro, via the simulated gastric and intestinal digestion of defatted walnut kernels with pellicle (DWKP) in a D-galactose (D-gal)-induced aging model in mice. Three doses were tested in parallel (75, 150, and 300 mg/kg bw/day) via intragastric administration for 6 weeks. DWKP hydrolysates (WKHs) showed significant antioxidant and anti-inflammatory effects against D-gal-induced changes in a dose-dependent manner: increased total antioxidant capacity (T-AOC) in all samples (*p* < 0.05 vs. the model group, MG), with complete restoring at the highest dose (*p* > 0.05 vs. control); augmented SOD activities in kidney and brain (*p* < 0.05 vs. MG), with complete recovering in all tissues and serum at the highest dose (*p* > 0.05 vs. control); and diminished MDA levels in tissues and sera of the aging mice at medium and high doses (*p* > 0.05 vs. MG), with complete recovery at the highest dose (*p* > 0.05 vs. control). Indeed, histopathological analysis proved that WKHs protect the tissue structure of the liver (prevent fat vacuole formation or venous obstruction) and kidney (prevent glomerular atrophy and nuclear aggregation). Immunohystochemistry revealed that inflammatory cytokine expression (TNF-α, IL-1β, and IL-6) was significantly reduced in the liver of D-gal-induced mice treated with WKHs, achieving with the highest dose the values comparable to those obtained in the control (*p* > 0.05 vs. control). The main active biomolecules identified in WKHs by UPLC-MS, responsible for these anti-aging effects, were polypeptides (50) and polyphenols (42), many of were hydrolysable tannins (23) belonging to the ET class [40].

##### Clinical Studies

According to multiple clinical investigations, Uro-A has been found to have the ability to slow down the aging process and enhance the treatment of age-related disorders affecting various organs like muscles, the brain, and skin [152]. Mitophagy, which represents the result of the degradation of mitochondria caused by exposure to exogenous inducers, occurs individuals age and in many age-related conditions [153]. Uro-A has been demonstrated to enhance muscle function and stimulate mitophagy in older animals, and induce mitochondrial gene expression in older humans [153]. Reestablishing appropriate levels of mitophagy is a potential technique with which to stop the decline of the organ functions associated with aging. Uro-A can start mitophagy and help damaged mitochondria be recycled through a process that depends on PTEN-induced kinase 1 (PINK1)/Parkin-dependent mitophagy, resulting in the selective ubiquitination of mitochondrial proteins for removal through phagolysosomal clearance [17].

In the context of DIRECT PLUS clinical trials, Meir et al. [49] investigated the impact of the green-MED diet on changes in biological age, specifically the correlations between dietary intake and changes in methylation age (mAge), as well as specific urine polyphenols. All mAge clocks were significantly correlated with the baseline chronological age (51.3 ± 10.6 years). The most significant attenuation of Li mAge (multivariate models adjusted for age, sex, baseline mAge, and weight loss) was primarily influenced by a higher intake of Mankai (*p* = 0.061) and green tea (*p* = 0.0016). This trend was associated with elevated levels of urine polyphenols, including hydroxytyrosol, tyrosol, Uro-C (*p* < 0.05 for all), and Uro-A (*p* = 0.08), which are abundant in green plants. A beneficial difference of about 8.9 months was seen between the observed and expected Li mAge at the end of the intervention (*p* = 0.02) for all subjects following MED-style diets [49].

#### 3.8.4. Antimicrobial Activity

Several investigations have examined the antimicrobial properties of walnut extracts rich in polyphenols. Using *Aspergilus flavus* NRRL 25347, a strain that produces aflatonin B1 (AFB1) preferentially, Mahoney and Molyneux [23] investigated which endogenous phytochemical elements have the capacity to prevent the synthesis of aflatoxins. They selected two walnut cultivars (cv.), Chico and Tulare, with high and low anti-aflatoxicogenic effects, respectively, based on a previous study. Aflatoxigenesis is triggered by the presence of cellular OS. The synthesis of aflatoxins is inhibited by hydrolysable tannins, particularly GA and gallotannins. Additionally, numerous natural polyphenolic compounds have been identified as antifungal agents. After successive walnut pellicle extractions in different solvents (hexane, acetone, methanol, and water), Mahoney and Molyneux quantified GA and EA in various fractions of the analyzed cultivars. They characterized the extracts by the EA/GA ratio and the inhibitory capacity of aflatoxigenesis in parallel with GA, EA, and tannic acid standards in *A. flavus* spore cultures. This was performed due to the remarkable antifungal activity of hydrolysable tannins. The Tulare cv. extracts in acetone, methanol, and water completely prevented the synthesis of AFB1 in comparison to the placement of the extract in hexane (which was undetectable) and residual ETs (78.6%). As compared to EA, which lowered aflatoxin levels to only 84% of control, GA reduced aflatoxin levels to 4% of those in the control. It was found that the formation of aflatoxins was not significantly influenced by EA, which also contains catechol groups that have the ability to complex metal ions and capture free radicals. Consequently, the primary component of WPE responsible for this activity is GA (not EA), and it acts to suppress the formation of AFB1 by pathways other than antioxidant action, most likely by regulating specific genes involved in the biosynthesis of AFB1 [23].

## 4. Strength, Limitations, and Future Prospects

Our study adds important knowledge in the field of nutrition and has specific strengths. This is the first systematic review to comprehensively examine the phytochemistry content, the bioavailability, metabolism, and bioactivity of ETs and their metabolites after walnut consumption, as well as their possible action mechanisms. The findings from preclinical and clinical studies emphasize that walnut ETs and their metabolites may have antioxidant and anti-inflammatory capacity, cardiometabolic and neuroprotective activities, and antitumor potential, and could prevent or reduce the impact of chronic and age-related diseases. Another significant strength was the use of information from heterogeneous reports from around the world. The majority of the included studies were carefully designed, conducted, and controlled, had appropriate sample sizes, and did not appear to have selection biases.

Although this review summarizes a wide range of preclinical and clinical studies, some limitations that could have prevented definitive conclusions should be considered: the quality of some studies was not even and several biases might have been present; relatively small sample sizes; a lack of uniform strains of murinic models; short intervention periods in several studies; the lack of a clearly defined GM; and various metabotypes could have led to adverse effects, such as OS-related pathologies when high concentrations of Uros end up exercising pro-oxidant activity. To overcome some of these problems, further preclinical and clinical experiments are needed to assess the preventive and therapeutic potential of walnut ETs and their metabolites: the use of animal models with humanized microbiota profile; in vivo experiments to analyze their toxicity; and the validation of animal results in human studies.

In walnut extracts, the ETs and EA metabolite group were shown to be the most representative of the phenolic antioxidants, totaling 85 compounds in the analytical studies. Despite the large number of phytochemical studies examined in this review, the most used method for the extraction of the phenolics was the conventional method, solid–liquid extraction. To increase the efficiency of the walnut extraction process, several innovative methods of assisted extraction should be developed in the future, including ultrasound technology, microwave technology, and pressurized/supercritical fluids, which present a series of advantages, such as reductions in extraction time, organic solvent consumption, or toxic residues, as well as higher yields.

Another limitation is the lack of diversity in Uro types. In the assessed clinical studies, the most analyzed Uros were Uro-A and its isomer, iso-Uro-A, as well as Uro-B, while the potential bioactivities of Uro-C, Uro-D, and Uro-M5 were previously only demonstrated in vitro studies [93]. For these reasons, it is important to investigate the biological activities of other types of Uros in preclinical and clinical studies in the future.

Moreover, due to poor absorption from the intestine, ETs and EA have a low bioavailability that limits their use and clinical efficacy. To overcome this limitation, a series of delivery systems and preparations have been proposed, such as EA inclusion complex-loaded hydrogels for orally controlled drug delivery [154,155] or the lipid nanoparticle-based formulation of EA (EA-liposomes) [156]. However, our research may further challenge the pharmaceutical industry to find new effective forms of conditioning EA and Uros, which would ensure optimal bioaccessibility and bioavailability with desirable therapeutic results.

In order to establish the optimal doses of ETs and EA, and, respectively, to evaluate the safety of ETs, EA and Uros, more pharmacokinetic studies are needed in humans, as is further exploration of the mechanisms of actions of these compounds. Finally, additional investigations are essential to understand the interindividual variability in response to the ingestion of ETs/EA-containing walnuts, and to elucidate the hypothesis that attributes the bioactivities derived from walnut consumption to Uros.

## 5. Conclusions

ETs, EA, and Uros have been shown to have a variety of beneficial effects, including anti-inflammatory, antioxidant, anticarcinogenic, antibacterial, cardioprotective, and neuroprotective properties, both in vitro and in vivo studies. Although recent evidence has shown the enhancement of mitochondrial and cellular health with urolithin ingestion in the elderly, data are still lacking in human studies. The health outcome contrast found in humans following the consumption of foods that are high in ETs can be largely attributed to the significant differences between individuals in the production of Uros resulting from GM variability. The beneficial effects are associated with the urolithin biosynthesis and the type of Uros that are produced. The primary metabolite detected in the plasma and urine of urolithin producers (UM-A and UM-B) is Uro-A. It has been linked to several health benefits, primarily the ability to reduce intestinal inflammation. Other bioactive metabolites with comparable effects include Uro-B and IsoUro-A, although these have received less research. 

The analyzed literature revealed that ETs, EA, and their metabolites have a positive impact on antioxidant and anti-inflammatory mediators, acting as singlet oxygen suppressors and hydrogen atom suppliers, eliminating free radicals, decreasing lipid peroxidation, and also decreasing the level of inflammatory markers (IFN-γ, IL-1β, IL-6, TNF-α, and sE-selectin). The results obtained both in vitro and in vivo supported the neuroprotective effects of walnut extracts containing ETs from the activation of the PKA/CREB/BDNF signaling pathway, but also on other action mechanisms, including the decreasing of the OS in cerebral tissues by regulating mitochondrial activity, the attenuation of cholinergic impairment,, the improvement of brain lesions, and the decreasing of neuroinflammatory biomarkers (TNF-α, IL-1β). Furthermore, evidence from reviewed studies associated walnut consumption with numerous other bioactivities, including hepatoprotective and anti-aging effects. 

In conclusion, our systematic review supports the preventive and therapeutic potential of walnut ETs and their metabolites generated by the GM. Walnuts are an important and valuable superfood, with implications in lifespan and healthspan, and should be present in the human diet as often as possible. Moreover, walnut extracts rich in polyphenolic compounds might be used in the pharmaceutical and food industry by inclusion in new medicinal preparations or food supplements. But for this, additional research is needed.

## Figures and Tables

**Table 1:** Characteristics of the selected studies.

Reference/ Country/ Study Type	Study Purpose/ Design	Type of Extracts/Biological Systems (Cell Lines, Animal Model, Clinical Trial)/Doses	Analysis Methods/ Tests/Biomarkers	Significantly Outcomes
[23] USA Analytical/ In vitro	WP phytochemical composition: GA, EA	WPE—MeOH, HCl	HPLC-UV	EA/GA ratio in WP: 4.4—Tulare cv. vs. 6.1—Chico cv.
	WP aflatoxigenicity inhibition potential	*A. flavus* NRRL 25347 spore suspension (aflatoxin B_1_); treatment: GA, EA, TA, WPE (hexane, acetone, MeOH, H_2_O)	HPLC-FD: Aflatoxin B_1_	WPE inhibit aflatoxigenesis ↓ Aflatoxin levels: GA to 4% of control (day 6) vs. EA to 84% of control (same day)
[24] Spain Clinical	Colonic microflora Uro-A production from EA, punicalagin, and ETs rich WE	Fecal samples (6 healthy donors); Treatment: EA, punicalagin, WKE	HPLC-DAD-MS/MS: 0, 5, 24, 48, 72 h	Identified: Uro-A in all cultures No correlation between daidzein and EA metabolisms by fecal microflora
[25] Spain Analytical/Clinical	ET composition of WK	WKE—80% MeOH	HPLC-DAD-ESI-MS/MS	Identified: 3 ETs: pedunculagin, valoneic acid dilactone, casuarictin
	ETs metabolism	Healthy volunteers (*n* = 40), WK: 35 g/day, single dose	HPLC-MS/MS, UV Urine: MeOH fractions (F1-F5)	Uro-B glucuronides: all urine fractions F3-F5 ETs, EA: ND Metabolite excretion: 16.6%
[26] Slovenia Analytical	Phenolic composition: WKs vs. WP	WKE—MeOH WPE—MeOH	HPLC-DAD	Syringic acid, juglone, EA (WKE, WPE): predominant—all cvs. WP: most important source of walnut phenolics
[27] China, Canada Analytical	Polyphenolic profiles, AAs: *J. ailanthifolia* L., *J. regia* L.	WKEs—80% MeOH WKEs—FPA, AHPA, and BPA fractions	HPLC-DAD, LC-ESI-MS Quantification: TPC AAs: FRAP, PCL assays	EA (FPA, AHPA, BPA) and valoneic acid dilactone (AHPA, BPA): Combe, Lake vars. TPC (FPA, AHPA): *J. regia* > *J. a.* L. FRAP and PCL values (FPA, AHPA, BPA): *J. r*. L. > *J. a.* L.
[28] Spain, Italy Analytical	CE-MS method developed to identify and quantify phenolic and related polar compounds in WK	WKE—80% MeOH	CE–ESI-TOF-MS	New ET: (2*E*,4*E*)-8-hydroxy-2,7-dimethyl-2,4-decadiene-1,10-dioic acid 6′-*O*-β-D-glucopiranosyl ester Gla A, Gla B, and the new ET: 72–86% of TAPC
[29] Greece Analytical/ In vitro	WE phytochemical composition	PWKE—MeOH	HPLC-DAD, TLC, NMR Quantification: TPC	Identified: EA, GA, catechin, caffeic acid, and coumaric acid TPC: 16.9 ± 0.8 μM MC/g dw
	AI activity in HAEC and osteoblastic activity in KS483 cells	HAEC cultures marked with TNF-α +/− WE (10, 50, 200 µg/mL) or EA (10^−7^–10^−5^ µM) 2KS483 osteoblastic cell cultures marked with ascorbic acid +/− WE (10, 25, 50 μg/mL) or EA (10^−9^–10^−6^ M)	Quantification: VCAM-1, ICAM-1 (ELISA) Cell viability: MTT Mineralized nodules: light microscopy	↓ VCAM-1 and ICAM-1 expressions vs. control WE and EA: ↑ nodule formation in KS483 osteoblasts
[30] USA In vitro	WKPhs and EA ability have to modulate cytokine levels and the cellular proliferation of stimulated human PBMCs	WKPhs: CE (24 h, 4 °C) HE (24 h, 56 °C) PBMC stimulation agents: PHA, α-CD3, or PMA/ionomycin	Cytokine levels: IL-2, IL-4, IL-13, TNF-α (ELISA kits) Proliferation assay: [3H]TdR incorporation, 24 h	Cytokine production from PHA-stimulated PBMCs: EA: ↑ IL-2 EA, CE, HE: ↓ IL-13 CE, HE: ↓ TNF-α IL-4: no changed WKPhs and EA: ↓ stimulated [PHA, α-CD3 or PMA/ionomycin] PBMC proliferation in a dose-dependent manner
[31] Spain Analytical/Clinical	ET composition of PWK	PWKE—80% MeOH	HPLC-DAD-MS/MS	Identified: EA and 10 ETs
	ETs, EA, and Uros identifying and quantifying in human prostate gland	PCa and BPH male patients (*n* = 14), PWK: 35 g/day, 3 days	HPLC-DAD-MS/MS: urine, plasma, prostate	Identified: Uro-A glucuronide, Uro-B glucuronide (traces), dimethyl ellagic acid Small number of prostates containing metabolites
	ETs, EA, and Uros effect on gene expression		Expression levels of CDKN1A, MKi-67 and c-Myc: prostate	Walnut ETs: no apparent effect on the expression of p21, c-Myc or MKi-67 in the prostate gland
[32] USA Clinical	Effect of walnut meal on metabolic profile	Healthy volunteers (*n* = 16), WK: 90 g/day, single dose	HPLC-UV, HPLC-MS: urine TPC: plasma	Uro-A (urine): ↑ following walnut meal GCG, ECG, EGCG (plasma): ↑ at 1 h
	Effect of a walnut meal on postprandial OS and antioxidants		AAs: FRAP, ORAC: plasma Lipid oxidation: MDA, oxidized LDL: plasma Lipidic profile, uric acid: serum	AUC0–5 h: ↓ MDA ↑ hydrophilic and lipophilic ORAC No change: total phenols, FRAP, uric acid ↓ Oxidized LDL at 2 h
[11] Spain Analytical	Screening the complete profile of WPhs	WKE—60% acetone	LC-ESI-LTQ-Orbitrap-MS Quantification: TPC AAs: ABTS+, DPPH assays	Identified: 120 compounds: hydrolysable/condensed tannins, flavonoids, phenolic acids (8 new walnut ETs) TPC: 2,464 ± 22 mg GAE/100 g ABTS^+^: 21.4 ± 2.0 mmol TE/100 g DPPH: 25.7 ± 2.1 mmol TE/100 g
[33] Spain Clinical	ETs and EA metabolism by human GM; urolithin phenotypes	Healthy volunteers (*n* = 20), WK: 30 g/day, 3 days	HPLC-DAD-ESI-IT-MS/MS: urine	Phenotype A: 65% Phenotype B: 20% Phenotype 0: 15%
[34] Spain Clinical	Uros chromatographic and spectroscopic characterization after ET and EA food intake	Healthy volunteers (*n* = 10), WK: 30 g/day, 3 days	HPLC-DAD-ESI-Q (MS) UPLC-ESI-QqQ (MS/MS) UPLC-ESI-QTOF(MS/MS) UV Urine, feces	Uros characterization: LC coupled to DAD and/or MS detectors (QqQ, QTOF) UV RRFs of different Uros compared with UV spectrum of Uro-A and EA: relevant for Uro quantification and identification
[35] Korea Analytical/ In vitro	WPhE: phytochemical composition	WPhE—50% MeOH	HPLC-PDA	Identified/quantified: EA, GA, (+)-catechin, chlorogenic acid
	Anti-CSC potential evaluation of WPhE and its bioactive compounds	CD133+CD44+ isolated from HCT116 cells and incubated with WPhE (0, 10, 20, and 40 μg/mL), (+)-catechin, chlorogenic acid, EA, and GA	Cell proliferation assay: MTT RT-PCR Western blot: protein expressions Clonogenic assay Sphere formation assay	WPhE: ↓ Cell growth, ↑ cytokeratin 20 (CK20) expression ↓ CD133, CD44, DLK1, Notch1, β-catechin, and p-GSK3β expressions, ↓ self-renewal CSCs capacity: colony formation and non-adherent spheroid formation
[36] Spain, Turkey Clinical	UMs identification	Healthy normoweight volunteers (*n* = 20), WK: 30 g/day, 3 days	UPLC-ESI-qToF-MS: urine	UM-A: 70% UM-B: 20%
	UMs and CMR factors		Lipidic/glycemic profile: plasma Bacterial DNA extraction, real-time qPCR, *Gordonibacter* spp.: feces	CMR factors and Uros: no correlations Fecal *Gordonibacter* correlations: Uro-A: positive Isouro-A + Uro-B: negative
[37] Spain, Belgium Clinical	UMs identification	Healthy volunteers (*n* = 27), PWK: 33 g/day, 3 days	UPLC-ESI-QTOF-MS: urine	Metabotype stratification: UM-A: Uro-A UM-B: Uro-B, IsoUro-A, Uro-A UM-0—no Uros
	UMs microbiota modulation		GM composition: 16S RNA illumina sequencing and qPCRs Microbial activity: SCFA analysis: feces	UM-B GM: sensitive to walnut intervention *Blautia, Bifidobacterium, Coriobacteriaceae* fam. members ↑ in UM-B *Lachnospiraceae* fam. members ↓ in UM-A *Coprococcus* and *Collinsella* ↑ in UM-A and UM-B Walnut: modulates GM in a UM-depending manner and ↑ SCFA production
[38] Spain Analytical/ Clinical	Free EA quantification in PWK	PWK—acid hydrolysis	HPLC-DAD-MS/MS	Identified/quantified: EA, primary precursor of Uros
	Uros identification in human breast milk	Healthy postpartum women (*n* = 27), PWK: 30 g/day, 3 days	HPLC-DAD-ESI-Q-MS: urine UPLC-ESI-QTOF MS: breast milk	Mothers UMs (urine): UM-A (44%), UM-B (55%); governed the breast milk urolithin profile Total Uros (breastmilk): 8.5–176.9 nM
	Kinetics of *Gordonibacter* colonization in newly born babies	Babies (*n* = 30) stool samples at 1, 4, 6, and 12 months	qPCR: *Gordonibacter:* breast milk, infant feces	Fecal *Gordonibacter* ↑ to 78% in 4-month-old babies from UM-A mothers, ↓ for 6-month-old babies from UM-B mothers Pattern of *Gordonibacter* in babies: conditioned by their mother’s UM
[39] Japan Analytical	Identification of characteristic component(s) in walnuts: quality evaluation	WKE—80% MeOH	NMR, LC-HR-ESI-MS/MS	Identified: 30 compounds New: Gla C, EA 4-*O*-(3′-*O*-galloyl)-β-D-xyloside, platycaryanin A methyl ester
[9] Slovenia Analytical	Identification and quantification of major phenolic constituents in WP and PWK	WPE—MeOH PWKE—MeOH	HPLC–MS/MS Quantification: TPC	Identified/quantified: 56 compounds WPE: 19 ETs, 12 EA derivatives, 4 anthocyanins, 5 other phenols (14 new) PWKE: 5 ETs, 10 dicarboxylic acid derivatives, 1 phenol (13 new) TPC: WP (~1000-fold) > PWK TPC intake/1 WK: highest Franquette, Rubina cvs.; lowest Krka cv.
[40] China Analytical/ In vivo	Identification of polypeptides and polyphenols in defatted WK	WK WKH: 1 M HCl, hydrolyzed: pepsin (E/S: 6/100, *w/w*), 37 °C for 1.0 h, tripsin (E/S: 1:25, *w/w*), 37 °C for 2.0 h	UPLC-Q-Orbitrap-MS Quantification: TPC (WK, WKH)	Identified 42 compounds: 13 ETs, 10 EA derivatives, 5 gallotannins, 1 ketone,1 flavanone, 2 esters, 2 flavonoids, 5 organic acids, 3 simple phenolic acids The major polyphenols: ETs TPC: WK: 4.90 mg GAE/g WKH: 40.17 mg GAE/g
	Protective effect of defatted walnut kernel hydrolysates (WKH, obtained by simulated GI digestion) in mice with d-gal-induced aging	SD mice, male—5 groups: normal (NG), model (MG), low-dose (WKH-L), medium-dose (WKH-M), and high-dose (WKH-H) groups Doses: 300 mg d-gal/kg bw/day (i.p.); WKH: 75, 150, and 300 mg/kg bw/day (i.g.) for 6 weeks	Biomarkers of OS: SOD, T-AOC, MDA in serum, liver, kidney, and brain tissues Histopathological analysis: liver and kidney Immunohistochemistry: TNF-α, IL-1β, and IL-6—liver	WKHs: recover T-AOC and SOD activity, and ↓ MDA in tissues and serum in d-gal-induced aging mice. WKH: protect the tissue structure of the liver and kidney and reduce the inflammatory biomarker expressions (TNF-α, IL-1β, and IL-6) in liver of mice with d-gal-induced aging
[41] China Analytical	Phenolic profiles and AAs of free, esterified and bound phenolic compounds in WK	PWKE—70% MeOH, PWKE—70% EtOH PWKE—70% acetone	UPLC-ESI-MS/MS Quantification: TPC, TFC AAs: DPPH, ABTS, TAC assays	EA, GA, ferulic acid, sinapic acid, caffeic acid: all forms EA: the major constituent: free form (7 times) > esterified form Walnut phenolics: free: 51.1–68.1%; bound: 21.0–38.0%; esterified: 9.7–18.7% Free phenolics: highest radical scavenging activity (IC_50_: DPPH, 15.5 µg/mL; ABTS, 13.6 µg/mL)
[42] Israel Clinical	WK metabolic profile	Healthy volunteers (*n* = 284), WK—28 g/day, 18 months (MED)	HPLC-QTOF: urine	Identified: Uro-A, tyrosol
	Effect of MED diet combined with physical activity on age-related brain atrophy		Lipidic profile, glycemia a jeun, HOMA-IR: plasma MRI-derived brain anatomical parameters: HOC and LVV	Participants ≥ 50 y of age: weight loss, lower HOMA-IR, and lower TG conc.: associated with lower decline in HOC. ↑ walnut consumption: associated with lower decline in HOC
[43] Korea Analytical/ In vitro/ In vivo	WKE phytochemical profile	WKE—80% EtOH	UPLC Q-TOF/MS	Identified: 2 EA derivatives, 4 ETs, and 1 flavanol
	Neuroprotective effect of WKE from GC on: (1) neuronal PC12 and hippocampal HT22 cell lines exposed to H_2_O_2_ or high glucose concentrations	PC12 and HT22 cell lines; H_2_O_2_: 200 μM; Glucose: 50 μM; WKE: 20 μg/mL and 50 μg/mL	Cell viability: MTT Intracellular ROS content: DCF-DA method	GC (20 and 50 μg/mL): - ↑ cell viability and ↓ ROS production in both cell lines
	(2) cognitive impairment in an animal model of HFD-induced diabetes	Male C57BL/6 mice—4 groups (*n* = 8): NC group (normal diet), HFD group (HFD for 12 weeks), GC20 and GC50 groups (HFD for 12 weeks + 20 and 50 mg/kg bw, respectively, orally, for 4 weeks)	Behavioral tests: Y-Maze, passive avoidance, and Morris Water Maze (MWM) tests Biochemical tests: LDH, TG, TC, HDL-c, LDL-c, HDL-c/TC ratio (HTR) OS biomarkers: FRAP and AGEs (serum) MDA (brain and liver) Cerebral cholinergic system: ACh level, AChE activity Mitochondrial activity in brain: ROS, MMP Western blot: protein expressions in brain	GC20 and GC50 restored the HFD-altered behavior: the ability, the step-through latency, the escape latency time, and the time in the W zone GC improved lipidic profile: ↓ total WAT and liver fat mass, ↓ LDL-c, ↓ LDH and TG vs. HFD GC50: - ↑ serum AA in FRAP assay and ↓ AGEs vs. HFD GC20 and GC50 attenuated cholinergic system impairment: ↑ ACh level, ↓ AChE activity,↓ AChE/β-actine, and ↑ ChAT/β-actine relative expressions vs. HFD GC20 and GC50: - ↓ mitochondrial ROS production and ↑ MMP levels in cerebral tissues vs. HFD - synergically regulated the *p*-JNK, *p*-Akt, p-tau, IDE, Aβ, BAX, and caspase-3 expressions vs. HFD ↓ neuroinflammation: ↓ protein expression of TNF-α, IL-1β, p-NFκB, caspase-1, and ↑ HO-1 expressions (*p* < 0.05 for all proteins for both GC20 and GC50 vs. HFD)
[44] China Analytical/ In silico/ In vitro/ In vivo	DWPE phytochemical profile	DWPE—80% EtOH	UPLC-Q-Exactive Orbitrap MS	Identified: 36 compounds: 9 new derivatives (dicarboxylic acid glycosides)
	Identification of the DWPE metabolites in rats	Male SD rats (*n* = 12): 10 g DWPE/kg (i.g.)	Metabolite profile: in rat plasma, bile, urine, and feces samples by UPLC-Q-Exactive Orbitrap MS	52 metabolites of DWPE identified in vivo (26 in plasma, 24 in bile, 36 in urine, and 13 in feces), derived from GA, EA and Gla A
	Pathway mechanism screening of DWPE metabolites against NAFLD and NASH by network pharmacology		Network pharmacology: Kyoto Encyclopedia of Genes and Genomes (KEGG) pathways	11 potential pathways identified, including inflammation, PPAR signaling, and nitrogen metabolism pathways
	Protective effect of DWPE against NAFLD: (1) on Oleic acid (OA)-induced HepG2 cells;	OA: 0.25 mM; DWPE: 25–200 μg/mL; L-ornitine L-aspartate (LOLA): 50 μM	Cell viabilities: at 25, 50, 100 and 200 μg/mL of DWPE; OA-induced cellular steatosis by Oil Red O staining; Western blot: protein expressions	DWPE (100 μg/mL): - ↓ intracellular lipid accumulation - ↓ intracellular ammonia concentration - ↑ CA2 and CPS1 expressions
	(2) in HFD-induced mice	Male C57BL/6 mice—5 groups (*n* = 8): (1) ND (normal diet); (2) HFD (HFD for 12 weeks); (3) DWPE-H and (4) DWPE-L (HFD + 1.2 g or 0.6 g DWEP/kg, respectively, orally, for 12 weeks); (5) LOLA (HFD + LOLA granules, 1.35 g/kg, orally, for 12 weeks)	Biochemical tests: TG, TC, ammonia levels (serum) Western blot: protein expressions in liver	- ↓ TG and TC levels in serum in DWPE-H group - ↓ ammonia concentrations in serum - ↑ CA2 and CPS1 expressions in liver
[45] Spain Clinical	UMs identification	PD patients (*n* = 52) and healthy controls (HC) (*n* = 117), WK: 30 g/day, 3 days	UPLC-ESI-QTOF-MS: urine	UM-A: PD—45%, HC—57% UM-B: PD—27%, HC—34% UM-0: PD—27%, HC—9% Significant ↑ of UM-0 as the disease severity ↑
	Uros as biomarkers of gut dysbiosis and stage disease in PD patients		GM composition, UMs: feces	GM of patients with UM-0 and highest severity PD: ↑ *Enterobacteriaceae* ↓ *Lachnospiraceae* members and *Gordonibacter*
[46] Israel Clinical	WK metabolic profile	Obese volunteers, BMI = 31.2 kg/m^2^ (*n* = 294) WK: 28 g/day, 18 months	HPLC-QTOF: urine	Identified: Uro-A
	Effect of MED diet on visceral adiposity		Magnetic resonance imaging (MRI)—to quantify the abdominal adipose tissues	MED diet: moderate weight loss (−2.7%), WC loss (−4.7%) MED diet VAT loss (−6.0%) Vs. green-MED-diet (−14.1%; *p* < 0.05) ↑ total plasma polyphenols (hippuric acid), Uro-A (urine)—significantly related to greater VAT loss (*p* < 0.05)
[47] China Analytical/ In vitro	WPhE phytochemical profile	WPhE—75% EtOH	UPLC-QTOF MS/MS	Identified: 13 phenolic compounds, of which 10 ETs (61.8% of TAPC, *w*/*w*)
	The neuroprotective effect of WPhE and Uro-A on H_2_O_2_-induced damage in SH-SY5Y cells and the mechanisms involving CREB signaling pathways	Human neuroblastoma SH-SY5Y cell cultures pretreated with WPhE (50–150 μg/mL) or Uro-A (2.5–20 μM) for 12 h and then exposed to H_2_O_2_ (200 μM)	Cell viability: MTT Cell apoptosis: by Hoechst 33342 staining Biochemical analysis: Extracellular LDH activity, intracellular Ca level, ROS, SOD, CAT Western blot analysis: protein expressions in the absence and in the presence of H89, a PKA inhibitor (pretreatment with 10 μM for 1 h)	Pretreatment with WPhE or Uro A: - protect SH-SY5Y cells viability against H_2_O_2_ damage: completely by >75 μg/mL WPhE; in a reversed ”U”-shape manner by Uro-A, with a maximum effect at 10 μM - ↓ number of apoptotic cells and normalize the nuclear chromatin morphology - ↓ (prevents) extracellular LDH leakage and intracellular Ca overload as well as ROS level - ↑ (prevents) SOD and CAT activities - ↑ cAMP-dependent PKA activity, pCREB (Ser133) and BDNF expressions PKA inhibitor H89 pretreatment: abolished the protective effects of WPhE and Uro-A
[48] China Analytical	Conversion of ETs into free EA in WKs during baking: investigating the EAC in the FPA, AHPA, and BPA fractions	WKE—60% acetone	LC-MS Quantification: EAC, ETC, and TPC	8 ETs: main precursors of EA in WK EAC in FPA: max. (5.17 ± 0.30 mg/g dw) after baking at 165 C for 30 min; ↑ by 99.52% compared to control. ETC in AHPA and BPA: ↓ by 89.14%, and 26.08% TPC: max. (102.29 ± 7.75 mg GAE/g dw) after baking at 150 °C for 30 min Baking: conversion of ETs in AHPA and BPA to EA in FPA
[49] Israel Clinical	WK metabolic profile	Healthy volunteers, abdominal obesity or dyslipidemia (*n* = 256), WK: 28 g/day, 8 months (MED)	HPLC-QToF: urine	Identified: Uro-A, Uro-C and hydroxytyrosol
	Effect of polyphenols on DNA methylation-assessed biological age attenuation		Biological aging epigenetic clocks: DNA methylation (Illumina EPIC array): blood	All interventions: did not differ in terms of changes between mAge clocks MED inversely associated with biological aging
[50] China Analytical	Identification of key antioxidants in free, esterified, and bound forms in WKs and WP	PWKE: 70% acetone WPE: 70% acetone	Phytochemical profile: UPLC-MS/MS Quantification: TPC AA: DPPH assay	Identified/quantified (PWKE, WPE): 31 phenolic compounds: phenolic acids, flavonoids, and 1 proanthocyanidin EA: the most abundant component in WKs (62.9%), and in WP (68.0%) BPA forms: WPE > PWKE TPC levels of all forms: positively correlated with AAs (R: 0.76–0.94, *p* < 0.05)
[51] China In vivo/ Molecular docking	Active fractions and substances of walnut kernel in a scopolamine-induced AD animal model	Male ICR mice—9 groups (*n* = 10): control, model (scopolamine, 3 mg/kg/day for 10 consecutive days), donepezil (positive, 0.65 mg/kg/day), WK, DWP, WO, WKP, WKOA, and WKPS (i.g. for 56 days, equivalent dose of 15.6 g crude W/kg)	Morris Water Maze Test Biochemical and ELISA: ACh, MDA, SOD, TNF-α, IL-6, IL-1 Histopathology analysis: hippocampus and cortex tissues Western blot: protein expressions in hippocampus and cortex	- ↓ escape latency time in WO, WKOA, and WKPS groups - ↑ attention time in WK, DWP and WKOA groups - ↑ spatial memory significant grater in WKOA group vs. other groups - WKOA and WKP restored Ach levels in hippocampus vs. model - WO, WKOA and WKP restored ACh levels in cerebral cortex vs. model - ↓ MDA levels in hippocampus and cortex by all WK fractions (but not WK) WKP and WKOA attenuated histopatological damage in scopolamine-induced AD mice brain - ↓ NFκB protein level in hippocampus in WKOA and WO groups as well as in cortex in WKOA group
	Distribution and metabolism of WKOA in brain tissue in AD model rat	Male SD rats (*n* = 6): control group and model group (scopolamine for 10 days) received WKOA (20x pharmaco-dynamic dose) on day 10	Metabolic profile in brain: UPLC-Q-Exactive Orbitrap MS	- 8 metabolites identified in rat brain after WKOA administration: p-hydroxycinnamic acid + 2H + sul; glansreginic acid + 2H + H_2_O; ellagic acid 4-*O* xyloside; ethyl gallate; EA; glansreginic acid; Gla A; ethyl gallate + sul
	Structure–activity relationship between the screened active compounds and AChE, BChE, SOD, IL-6, IL-1β, and TNF-α		Molecular docking: Surflex-Dock Geom (SFXC)	Main function: Gla A, glansreginic acid, and glansreginic acid + 2H + H_2_O as AChE and BChE inhibitors; EA and ellagic acid 4-*O* xyloside as OS and neuroinflammation inhibitors

AA—antioxidant activity; ABTS—2,2′-azino-bis(3-ethylbenzothiazoline-6-sulfonic acid); AChE—acetylcholinesterase; AD—Alzheimer’s disease; AGEs—advanced glycation end products; AHPA—acid-hydrolysable phenolic acid; AI—anti-inflammatory; ApoA-I—apolipoprotein A-I; ApoB—apolipoprotein B; BChE—butyrylcholinesterase; BDNF—brain-derived neurotrophic factor; BPA—bound phenolic acid; BPH—benign prostatic hyperplasia; CA2—carbonic anhydrase 2; CAT—catalase; CE—cold extract; CE-MS—capillary electrophoresis mass spectrometry; ChAT—choline acetyltransferase; CPS1—carbamoylphosphate synthetase; CREB—cAMP-response element binding protein; CRF—cardiometabolic risk factors; CSCs—cancer stem cells; CT—clinical trial; CTC—condensed tannin content; cv.—cultivar; DAD—diode array detector; DCF-DA—2′,7′-dichlorodihydrofluorecein diacetate; DIRECT PLUS—Dietary Intervention Randomized Controlled Trial Polyphenols Unprocessed Study; DPPH—2,2-diphenyl-1-picrylhydrazyl; dw—dry weight; DWP—defatted walnut powder; DWPE—defatted walnut powder extract; EA—ellagic acid; EAC—ellagic acid content; ECG—epicatechin gallate; EGCG—epigallocatechin gallate; ESI—electrospray ionization source; ETC—ellagitannin content; EtOH—ethanol; ETs—ellagitanins; FD—fluorescence detector; FPA—free phenolic acid; FRAP—ferric reducing antioxidant power; GA—gallic acid; GAE—gallic acid equivalents; GC—Gim-cheon 1ho cultivar; GC/MS—gas chromatography–mass spectrometry; GCG—gallocatechin gallate; GI—gastrointestinal; Gla—Glansreginin; GM—gut microbiota; green-MED diet—Mediterranean diet higher in polyphenols and lower in red/processed meat; HAEC—human aorta endothelial cells; HC—healthy control; HDL-c—high-density lipoprotein cholesterol; HE—heat extract; HFD—high-fat diet; HOC—hippocampal occupancy score; HOMA-IR—Homeostatic Model Assessment for Insulin Resistance; HPIC—high-performance ion chromatography; HPLC—high-performance liquid chromatography; HR—high resolution; ICAM-1—intracellular adhesion molecule 1; IDL-c—intermediate lipoprotein cholesterol; i.g.—intragastrically; IL—interleukin; IsoUro-A—isourolithin A; LC—liquid chromatography; LDH—lactate dehydrogenase; LDL-c—low-density lipoprotein cholesterol; LPS—lipopolysaccharide; LTQ—linear trap quadrupole; LVVc—lateral ventricle volume; mAge—methylation-assessed biological age; MC—4-methyl-catechol; MDA—malondialdehyde; MED diet—Mediterranean diet; MeOH—methanol; MetS—metabolic syndrome; MMP—mitochondrial membrane potential; MS—mass spectrometry; MTT—3-(4,5-dimethylthiazol-2-yl)-2,5-diphenyltetrazolium bromide; NAFLD—non-alcoholic fatty liver disease; NMR—nuclear magnetic resonance; PBMCs—peripheral blood mononuclear cells; PCa—prostate cancer; PCL—photochemiluminesence; PD—Parkinson’s disease; PDA—photodiode array detector; PHA—phytohemagglutin; PKA—protein kinase A; PMA—phorbol myristate acetate; PWK—peeled walnut kernel; PWKE—peeled walnut kernel extract; Q (MS)—single quadrupole mass spectrometer detector; qPCRs—specific real-time quantitative polymerase chain reactions; QqQ-MS—triple quadrupole mass spectrometry; RCT—randomized controlled trial; ROS—reactive oxygen species; RRFs—relative response factors; RT-PCR—reverse transcriptase polymerase chain reaction; SCFA—short-chain fatty acids; SOD—superoxide dismutase; TA—tannic acid; TAPC—total analyzed phenolics content; TE—Trolox equivalents; TFC—total flavonoid content; TLC—thin-layer chromatography; TOF—time of flight; TPC—total phenolic content; UM—urolithin metabotypes; UPLC—ultra-performance liquid chromatography; Uro-A—urolithin A; Uro-B—urolithin B; Uro-C—urolithin C; Uros—urolithins; vars.—varieties; VAT—visceral adipose tissue; VCAM-1—vascular cell adhesion molecule 1; WAT—white adipose tissue; WE—walnut extract; WK—walnut kernel; WKE—walnut kernel extract; WKH—walnut kernel hydrolysate; WKOA—walnut kernel organic acid; WKP—walnut kernel protein; WKPhs—walnut kernel polyphenols; WKPSs—walnut kernel polysaccharides; WO—walnut kernel oil; WP—walnut pellicle; WPE—walnut pellicle extract; WPhs—walnut polyphenols; WPhE—walnut polyphenol extract; ↓—decreased; ↑—increased.

**Table 2:** Identification and quantification of ellagic acid and ellagitannins in walnut (*J. regia* L.) kernels and pellicles.

Ref.	Extract	Analytical Method	Compounds	Amount			
[23]	Methanolic HCl extract of WP—8 walnut varieties:	HPLC-DAD					
	Chandler		Ellagic acid	10.0 ± 0.50 g/100 g dw			
	Chico		Ellagic acid	11.0 ± 0.70 g/100 g dw			
	Serr		Ellagic acid	11.8 ± 0.03 g/100 g dw			
	Payne		Ellagic acid	12.3 ± 0.10 g/100 g dw			
	Hartley		Ellagic acid	13.3 ± 0.10 g/100 g dw			
	Tehama		Ellagic acid	11.0 ± 0.15 g/100 g dw			
	Tulare		Ellagic acid	14.0 ± 0.15 g/100 g dw			
	Red Zinger		Ellagic acid	15.9 ± 0.20 g/100 g dw			
[24]	80% methanolic extract of WK	HPLC-DAD-ESI-MS/MS	Casuarictin				
			Pedunculagin	NQ			
			Valoneic acid dilactone				
[26]	Methanolic extracts of WKs and WP—10 walnut cultivars:	HPLC-DAD		WK		WP	
	Cisco		Ellagic acid	6.70 ± 0.60 mg/100 g		128.71 ± 6.73 mg/100 g	
	Fernette		Ellagic acid	3.26 ± 0.18 mg/100 g		60.66 ± 6.28 mg/100 g	
	Ferner		Ellagic acid	4.17 ± 0.35 mg/100 g		89.34 ± 3.80 mg/100 g	
	Rasna		Ellagic acid	6.59 ± 0.53 mg/100 g		124.46 ± 6.05 mg/100 g	
	A-117		Ellagic acid	9.77 ± 0.75 mg/100 g		266.19 ± 10.98 mg/100 g	
	Franquette		Ellagic acid	8.87 ± 0.91 mg/100 g		200.08 ± 3.08 mg/100 g	
	Adams		Ellagic acid	5.75 ± 0.60 mg/100 g		118.25 ± 1.86 mg/100 g	
	Lara		Ellagic acid	4.53 ± 0.31 mg/100 g		94.03 ± 1.97 mg/100 g	
	Chandler		Ellagic acid	4.30 ± 0.26 mg/100 g		78.36 ± 2.29 mg/100 g	
	Elit		Ellagic acid	5.09 ± 0.56 mg/100 g		129.73 ± 3.19 mg/100 g	
[27]				FPA		AHPA	BPA
	80% methanolic extract of defatted WK—Combe variety	HPLC-DAD, LC-ESI-MS^n^	Ellagic acid	0.32 mg/g of nut		1.30 mg/g of nut	1.21 mg/g of nut
	80% methanolic extract of defatted WK—lake variety		Ellagic acid	0.25 mg/g of nut		1.33 mg/g of nut	0.64 mg/g of nut
[28]	80% ethanolic extract of defatted WK—3 walnut varieties:	CE–ESI-TOF-MS	*Ellagic acid derivatives*				
	Chandler		Ellagic acid	24.7 ± 2.1 mg/kg dw			
	Howard		Ellagic acid	12.4 ± 0.3 mg/kg dw			
	Hartley		Ellagic acid	6.9 ± 1.3 mg/kg dw			
	Chandler		Ellagic acid pentoside dimer	36.1 ± 3.6 mg/kg dw			
	Howard		Ellagic acid pentoside dimer	33.0 ± 3.9 mg/kg dw			
	Hartley		Ellagic acid pentoside dimer	37.2 ± 3.1 mg/kg dw			
			*Ellagitannins*				
	Chandler		Glansreginin A	76.3 ± 15.6 mg/kg dw			
	Howard		Glansreginin A	335.6 ± 22.9 mg/kg dw			
	Hartley		Glansreginin A	76.3 ± 14.3 mg/kg dw			
	Chandler		Glansreginin B	92.1 ± 30.6 mg/kg dw			
	Howard		Glansreginin B	35.5 ± 5.5 mg/kg dw			
	Hartley		Glansreginin B	99.7 ± 0.1 mg/kg dw			
	Chandler		(2*E*,4*E*)-8-hydroxy-2,7-dimethyl-2,4-decadiene-1,10-dioic acid 6′-*O*-β-D-glucopiranosyl ester	40.8 ± 6.21 mg/kg dw			
	Howard		(2*E*,4*E*)-8-hydroxy-2,7-dimethyl-2,4-decadiene-1,10-dioic acid 6′-*O*-β-D-glucopiranosyl ester	48.3 ± 4.3 mg/kg dw			
	Hartley		(2*E*,4*E*)-8-hydroxy-2,7-dimethyl-2,4-decadiene-1,10-dioic acid 6′-*O*-β-D-glucopiranosyl ester	32.3 ± 3.9 mg/kg dw			
[29]	Methanolic extract of peeled, defatted WK	TLC, NMR, HPLC-DAD	Ellagic acid	NQ			
[31]	80% methanolic extract of peeled WK	HPLC-DAD-MS/MS	Ellagic acid	NQ			
			*Ellagitannins*				
			Casuarictin				
			Glansrin A				
			Glansrin B				
			Glansrin C				
			Pedunculagin				
			Rugosin				
			Stenophyllanin A				
			Tellimagrandin I				
			Tellimagrandin II				
			2,3-hexahydroxydiphenoyl-b-D-glucopyranoside				
[11]	60% acetone–water extract of WK	LC-ESI-LTQ-Orbitrap-MS	*Ellagic acid derivatives*	NQ			
			Ellagic acid				
			Ellagic acid pentoside isomer				
			Ellagic acid hexoside (2 isomers)				
			*Ellagitannins*				
			Alienanin B (3 isomers)				
			Casuarinin/casuarictin (2 isomers)				
			Euprostin A (2 isomers)				
			Eucalbanin A/cornusiin B (3 isomers)				
			Glansreginin A				
			Glansreginin B				
			Glansrin B (3 isomers)				
			Glansrin C (4 isomers)				
			Glansrin D/degalloyl rugosin F (3 isomers)				
			Heterophylliin D				
			Heterophylliin E (2 isomers)				
			HHDP-glucose (3 isomers)				
			Malabathrin A isomer				
			Oenothein B (2 isomers)				
			Pedunculagin/casuariin (bis-HHDP-glucose) (4 isomers)				
			Praecoxin A/platycariin isomer (trigalloyl-HHDP-glucose) (5 isomers)				
			Pterocarinin A (2 isomers)				
			Pterocarinin B				
			Reginin A/reginin D isomer (5 isomers)				
			Rugosin C/platycaryanin A/glansrin A (3 isomers)				
			Rugosin F				
			2′,3′-bis-*O*-degalloyl rugosin F isomer				
			1,2′,3′-tris-*O*-degalloyl rugosin Fisomer				
			Stenophyllanin A/B (2 isomers)				
			Stenophyllanin C (2 isomers)				
			Strictinin/isostrictinin (galloyl-HHDP-glucose) (6 isomers)				
			Tellimagrandin I (digalloyl-HHDP-glucose) (5 isomers)				
			Tellimagrandin II/pterocaryanin C (2 isomers)				
			Valoneic acid dilactone/sanguisorbic acid dilactone (2 isomers)				
[35]	50% methanolic extract of WK	HPLC-PDA	Ellagic acid	12.6 mg/100 g			
[38]	Peeled WK	HPLC-DAD-MS/MS	Ellagic acid	4.1 ± 0.6 mg/g fw			
[39]	80% methanolic extract of WK	NMR, LC-HR-ESI-MS/MS	*Ellagic acid derivatives*	NQ			
			Ellagic acid				
			Ellagic acid 4-*O*-β-D-xyloside				
			Ellagic acid 4-*O*-(3′-*O*-galloyl)-β-D-xyloside				
			*Ellagitannins*				
			Casuarictin				
			Casuarinin				
			Euprostin A				
			Glansreginin A				
			Glansreginin B				
			Glansreginin C				
			Glansreginic acid				
			Glansreginic acid 8-*O*-β-D-glucoside				
			Isostrictinin				
			Pedunculagin				
			Platycaryanin A methyl ester				
			Pterocarinin C				
			Rugosin C				
			Rugosin C methyl ester				
			Strictinin				
			Tellimagrandin I				
			Tellimagrandin II				
			Valoneic acid dilactone methyl ester				
[9]	Methanolic extract of WP	HPLC-MS/MS	*Ellagic acid derivatives*				
			Ellagic acid	17.5–23.3 mg/g fw			
			Ellagic acid pentoside	27.3–37.2 mg/g fw			
			Galloyl ellagic acid derivative	10.9–14.1 mg/g fw			
			Ellagic acid derivative 1	6.4–12.0 mg/g fw			
			Ellagic acid derivative 2	15.7–20.3 mg/g fw			
			Ellagic acid derivative 3	24.4–35.9 mg/g fw			
			Ellagic acid derivative 4	9.9–16.6 mg/g fw			
			Ellagic acid derivative 5	12.4–21.0 mg/g fw			
			Ellagic acid derivative 6	27.1–46.3 mg/g fw			
			Ellagic acid derivative 7	13.4–21.1 mg/g fw			
			Ellagic acid derivative 8	11.4–17.7 mg/g fw			
			Ellagic acid derivative 9	7.3–10.5 mg/g fw			
			*Ellagitannins*				
			bis-HHDP-glucose derivative	20.2–26.1 mg/g fw			
			Castalagin/vescalagin isomer 1	17.8–25.4 mg/g fw			
			Castalagin/vescalagin isomer 2	22.1–35.9 mg/g fw			
			Castalagin/vescalagin isomer 3	9.5–15 mg/g fw			
			Casuarin/casuarictin isomer (galloyl-bis-HHDP glucose) 1	23.9–39.8 mg/g fw			
			Casuarin/casuarictin isomer (galloyl-bis-HHDP glucose) 2	8.6–18.7 mg/g fw			
			Pedunculagin/casuariin isomer (bis-HHDP-glucose) 1	6.5–13.3 mg/g fw			
			Pedunculagin/casuariin isomer (bis-HHDP-glucose) 2	3.1–5.8 mg/g fw			
			Pterocarinin A isomer	0.5–2.8 mg/g fw			
			Strictinin/isostrictinin isomer (galloyl-HHDP-glucose) 1	1.9–2.9 mg/g fw			
			Strictinin/isostrictinin isomer (galloyl-HHDP-glucose) 2	7.3–9.6 mg/g fw			
			Strictinin/isostrictinin isomer (galloyl-HHDP-glucose) 3	7.0–9.5 mg/g fw			
			Tellimagrandin 1 isomer (digalloyl-HHDP-glucose) 1	6.2–10.1 mg/g fw			
			Tellimagrandin 1 isomer (digalloyl-HHDP-glucose) 2	3.1–14.1 mg/g fw			
			Tellimagrandin 1 isomer (digalloyl-HHDP-glucose) 3	18.4–27.9 mg/g fw			
			Trigalloyl-HHDP-glucose isomer 1	5.7–7.9 mg/g fw			
			Trigalloyl-HHDP-glucose isomer 2	2.9–4.0 mg/g fw			
			Trigalloyl-HHDP-glucose isomer 3	3.2–4.2 mg/g fw			
			Trigalloyl-HHDP-glucose isomer 4	17.8–27.5 mg/g fw			
	Methanolic extract of peeled WK		*Ellagitannins*				
			Glansreginin A	103.0–846.7 mg/kg fw			
			Glansreginin A [M+2H]	11.4–24.9 mg/kg fw			
			Glansreginin B	84.1–175.8 mg/kg fw			
			Glansreginin B [M+2H]	4.0–14.1 mg/kg fw			
			Glansreginin B hexoside	13.7–32.6 mg/kg fw			
[40]	WK hydrolysates	UPLC-Q-Orbitrap-MS	*Ellagic acid derivatives*			NQ	
			Ellagic acid				
			Ellagic acid pentoside				
			Ellagic acid hexoside				
			Ellagic acid-acetylglucoside				
			Ellagic acid diglycoside				
			Ellagic rhamnoside (2 isomers)				
			Methyl ellagic acid glucoside				
			Dimethyl ellagic acid				
			3,4-*O*, *O*-methylene-3′, 4′-*O*-dimethyl ellagic acid				
			*Ellagitannins*				
			Casuarinin/casuarictin				
			Glansreginin A				
			Glansreginin B				
			Glansrin C (2 isomers)				
			HHDP-glucose				
			Pedunculagin/casuariin (bis-HHDP-glucose) (3 isomers)				
			Strictinin/isostrictinin (galloyl-HHDP-glucose) (2 isomers)				
			Tellimagrandin (digalloyl-HHDP-glucose) (2 isomers)				
[41]		UPLC-ESI-MS/MS		Free		Esterified	Bound
	70% methanol/water extract of peeled, defatted WK		Ellagic acid	100.085 μg/g dw		7.518 μg/g dw	93.275 μg/g dw
	70% ethanol/water extract of peeled, defatted WK		Ellagic acid	112.711 μg/g dw		10.073 μg/g dw	79.801 μg/g dw
	70% acetone/water extract of peeled, defatted WK		Ellagic acid	146.331 μg/g dw		66.376 μg/g dw	46.380 μg/g dw
[43]	80% ethanolic extract of WK	UPLC IMS Q-TOF/MS	*Ellagic acid derivatives*	NQ			
			Ellagic acid				
			Ellagic acid-*O*-pentoside				
			*Ellagitannins*				
			Pedunculagin/casuariin isomer (bis-HHDP-glucose) I				
			Pedunculagin/casuariin isomer (bis-HHDP-glucose) II				
			Strictinin				
			Tellimagrandin I (digalloyl-HHDP-glucopyranose)				
[44]	80% ethanolic extract of defatted WK	UPLC-Q-Exactive Orbitrap MS	*Ellagic acid derivatives*	NQ			
			Ellagic acid				
			Ellagic acid hexoside				
			Ellagic acid 4-*O*-xyloside				
			3-*O*-methylellagic acid-pentoside				
			*Ellagitannins*				
			Glansreginin A				
			Glansreginin A+2H				
			Glansreginin A-H_2_O				
			Glansreginin A-H_2_O-2H				
			Glansreginin A+H_2_O+2H				
			Glansreginin A+Glc				
			Glansreginin B				
			Glansreginin B+2H				
			Glansreginin B-H_2_O				
			Glansreginin C				
			Glansreginic acid+2H				
			Glansreginic acid 8-*O*-β-D-glucoside				
			HHDP-glucose (2 isomers)				
			Pedunculagin/casuariin				
			Strictinin/isostrictinin (2 isomers)				
			Valoneic acid dilactone				
[47]	75% ethanolic extract of defatted WK		Ellagic acid	NQ			
		UPLC-Q-TOF MS/MS	*Ellagitannins*				
			Casuarinin/casuarictin isomer (2 isomers)				
			Glansrin B isomer				
			Glansrin C isomer				
			Pedunculagin/casuariin (2 isomers)				
			Praecoxin A/platycariin isomer				
			Sanguisorbic acid dilactone (2 isomers)				
			Strictinin/isostrictinin (2 isomers)				
			Tellimagrandin I (4 isomers)				
			Valoneic acid dilactone (2 isomers)				
[48]	60% acetone–water extracts defatted WK	LC-MS	*Ellagic acid derivatives*	NQ			
			Ellagic acid				
			Ellagic acid pentoside isomer				
			Ellagic acid hexoside (HHDP-hexoside)				
			*Ellagitannins*				
			Casuarinin/casuarictin (galloyl-bis-HHDP-glucose) (2 isomers)				
			Glansrin C(trigalloyl-HHDP-glucose) (2 isomers)				
			HHDP-glucose (2 isomers)				
			Pedunculagin/casuariin (bis-HHDP-glucose) (2 isomers)				
			Praecoxin A/platycariin (trisgalloyl-HHDP-glucose) (2 isomers)				
			Strictinin/isostrictinin isomer (galloyl-HHDP-hexoside)				
[50]				Free	Esterified	Bound	Total
	70% acetone–water extracts of peeled, defatted WKs	UPLC-MS/MS	Ellagic acid	56.49–164.95 μg/g dw	5.83–28.10 μg/g dw	0.82–5.58 μg/g dw	109.88 μg/g dw
	70% acetone–water extracts of WS		Ellagic acid	448.15–929.34 μg/g dw	600.99–724.70 μg/g dw	254.32–602.69 μg/g dw	1666.90 μg/g dw

AHPA—acid-hydrolysable phenolic acid; BPA—bound phenolic acid; CE—capillary electrophoresis; DAD—diode array detector; dw—dry weight; ESI—electrospray ionization; FPA—free phenolic acid; fw—fresh weight; HHDP—hexahydroxydiphenic acid; HPLC—high-performance liquid chromatography; HR—high resolution; IMS—ion mobility separation; LC—liquid chromatography; LTQ—linear trap quadrupole; MS—mass spectrometry; MSn—multi-stage mass spectrometry; NMR—nuclear magnetic resonance; NQ—non-quantified; PDA—photodiode array detector; Q—quadrupole; TLC—thin-layer chromatography; TOF—time of flight; UPLC—ultra-performance liquid chromatography; WK—walnut kernel; WP—walnut pellicle; WS—walnut skin.

**Table 3:** Metabolic compounds derived from ETs and EA after walnut *(J. regia* L.) intake, identified and quantified in different biological samples.

Study Type/ Reference	Study Design/Biological Cultures, Animal Model, Participants	Walnut Treatment/ Control	Biological Material Analyzed/ Samples Extracts Type	Analytical Method	Compounds/ Amount/ Metabotypes
In vitro [25]	6 healthy donors feces age: 25–30 y	Walnut extracts: EA:10 µg/mL ETs: 110 μg EA Eq/mL Punicalagin: 100 µg/mL Control: Daidzein: 1 g/mL	*Feces samples:* microflora cultures; diethyl ether	HPLC-DAD-MS/MS	Uro-A (48 h) *EA extract:* V2: 294.81 µg/100 mL fecal culture V3: 620.65 µg/100 mL fecal culture *ET extract:* V2: 386.37 µg/100 mL fecal culture V3: 321.55 µg/100 mL fecal culture
In vivo [44]	Male SD rats (*n* = 12), 180–220 g	DWPE: 10 g/kg (i.g.)	*Plasma samples:* (0, 1, 2, 4, 8, 10, 12, 24, 36, 48 h); MeOH extract *Bile samples*: (12 h); MeOH, 0.1% formic acid in H_2_O *Urine and feces samples:* (12 h), MeOH	UPLC-Q-Exactive Orbitrap MS	Ellagic acid—NQ Glansreginin A—NQ Glansreginic acid—NQ Glansreginic acid 8-O-β-D-glucoside—NQ Uro-M5—NQ Uro-D—NQ Uro-C—NQ
In vivo [51]	Male ICR mice (*n* = 10), 9 groups	WK, DWP, WO, WKP, WKOA, and WKPS: 15.6 g kg/day (i.g.), 56 days Control model: Scopolamine: 3 mg/kg/day, 10 days Donepezil: 0.65 mg/kg/day	*Brain samples:* 0.1% ACN, MeOH	UPLC-Q-Exactive Orbitrap MS	*WKOA:* p-Hydroxycinnamic acid + 2H + sul—NQ Glansreginic acid + 2H + H_2_O—NQ Ellagic acid 4-*O* xyloside—NQ Ethyl gallate—NQ EA—NQ Glansreginic acid—NQ Glansreginin A—NQ Ethyl gallate + sul—NQ
Clinical [24]	CT: 40 healthy (20 females), Age (mean): 29 y	WK—35 g/day (191 mg EA), 1 dose Control: ET-free diet	*Urine samples:* (8, 16, 32, 40, 56 h), fractions F1-F5, MeOH	HPLC-ESI- MS/MS, UV HPLC–APCI–MS/MS, UV	F1→F5: ETs, EA—ND Uro-B gluc: F1: ND F2: <LOQ F3: 8.3 ± 18.4 mg F4: 10.7 ± 20.4 mg F5: 12.6 ± 14.5 mg
Clinical [31]	RCT: 14 PCa and BPH male patients, age: 68.9 ±7.6 y (56–90)	PWK—35 g/day, 3 days Control: ET-free diet	*Urine samples:*	HPLC-DAD-MS/MS, UV	Uro-A gluc: 9 patients > 5 µM, 4 patients < 5 µM, 1 patient <LOQ
			*Plasma samples*: MeOH		Uro-A gluc: 0.11±0.05 mM Uro-C gluc—NQ Uro-C methyl ether glucuronide—NQ Dimethyl ellagic acid glucuronide—NQ
			*Prostate samples*: MeOH: HCl:H_2_O (79,9:0,1:20, *v*:*v*:*v*), MeOH		Uro-A gluc: 0.5–2 ng/g Uro-B gluc—NQ Dimethyl ellagic acid glucuronide—NQ
Clinical [32]	RCT: 16 healthy (10 females), age: 26 y (23–44)	WK—90 g/day, 1 dose Control: ET-free diet	*Urine samples:* (0–12 h, 12–24 h), 2% formic acid/MeOH (9:1 *v*/*v*); diethyl ether Enzymatic treatement: β-glucoronidase + sulfatase	HPLC-UV HPLC-MS	0–12 h/12–24 h: 3,4-Dihydroxyphenylacetic acid: 0.375 ±197 mM/0.401 ± 210 mM 4-Hydroxyphenylacetic acid: 48.90 ± 25.37 mM/72.44 ± 36.15 mM 4-Methoxyphenylacetic acid: 4.59 ± 1.82 mM/7.71 ± 1.70 mM (*p* < 0.05) Uro-A: 20.44 ± 32.18 μM/100.59 ± 114.86 μM
			*Plasma samples:* (1 h, 2 h), MeOH		LS Mean, 1 h/2 h: Gallocatechin gallate: 4.63 ng/mL (*p* < 0.05)/1.33 ng/mL Epicatechin gallate: 12.77 ng/mL (*p* < 0.05)/6.18 ng/mL Epigallocatechin gallate: 108.6 ng/mL (*p* < 0.05)/26.60 ng/mL
Clinical [33]	CT: 20 healthy (9 females), age: 21–55 y	WK- 30 g/day (162.8 mg EA), 3 days Control: ET-free diet	*Urine samples:* 0.1% formic acid in H_2_O	HPLC-DAD-ESI-IT-MS/MS, UV	UM-A—65% UM-B—20% UM-0—15%
Clinical [34]	CT: 10 healthy, age: 21–55 y	PWK—30 g/day, (5.1 mg free EA/g), 3 days Control: ET-free diet	*Urine samples:* 0.1% formic acid in H_2_O	HPLC-DAD-ESI-Q (MS) UPLC-ESI-QqQ (MS/MS) UPLC-ESI-QTOF (MS/MS) HPLC-UV	*Urine ^a^* (mean ± SD): Uro-A 3-gluc: 70.0 ± 57.4 mg/24 h IsoUro-A 3-gluc: 34.3 ± 36.3 mg/24 h Uro-B gluc: 88.8 ± 1.2 mg/24 h IsoUro-A: 1.0 ± 0.60 mg/24 h Uro-A: 1.7 ± 1.5 mg/24 h Uro-B: 1.0 ± 0.6 mg/24 h
			*Feces samples:* 0.1% HCl in MeOH:H_2_O (80:20, *v*/*v*), MeOH		*Feces ^b^* (mean ± SD): Uro-D: 6.4 ± 1.4 μg/g Uro-M6: 17.2 ± 7.3 μg/g Uro-C: 74.4 ± 88.0 μg/g Uro-M7: 49.9 ± 38.4 μg/g IsoUro-A: 217.8 ± 291.9 μg/g Uro-A: 121.2 ± 85.0 μg/g Uro-B: 108.6 ± 155.4 μg/g
Clinical [36]	CT: 20 healthy normoweight (9 females), age: 33.6 ± 10.2 y	PWK—30 g/day, 3 days Control: Pomegranate extract: 450 mg/day Nuts: 15 g-walnuts, 7.5 g-hazelnuts, 7.5 g-almonds/day	*Urine samples:* 0.1% formic acid in H_2_O	UPLC-ESI-QTOF MS	UM-A: normoweight (70%), overweight–obese (57%), MetS (50%) UM-B: normoweight (20%), overweight–obese (31%), MetS (41%)
Clinical [37]	CT: 27 healthy, (15 females), age: 39.5 ± 7.3 y	PWK—33 g/day, 3 days Control: ET-free diet	*Urine samples:* 0.1% formic acid in H_2_O	UPLC-ESI-QTOF-MS	UM-A—52% UM-B—48% UM-0—0%
Clinical [38]	*Trial 1*—Pilot study: 11 healthy postpartum women, *Trial 2*—CT: 27 healthy postpartum women	PWK—30 g/day, 3 days Control: ET-free diet	*Breast milk samples:* (24, 48, 72 h), ACN/formic acid (99:1, *v*/*v*), MeOH	UPLC-ESI-QTOF-MS	*Trial 1* Uro-A, IsoUro-A, Uro-B, Uro-A gluc, IsoUro-A gluc, Uro-A sulfate, Uro-B gluc, Uro-B sulfate Total urolithins: 8.5–176.9 nM *Trial 2* Uro-A gluc—UM-A: 27.6 ± 22.9 nM; UM-B: 31.1 ± 24.8 nM Uro-A sulfate—UM-A: 7.9 ± 1.8 nM; UM-B: 17.2 ± 11.8 nM IsoUro-A gluc—UM-A: <LOD; UM-B: 14.8 ± 8.6 nM Uro-B gluc—UM-A: <LOD; UM-B: 19.8 ± 21.6 nM Uro-B sulfate—UM-A: <LOD; UM-B: <LOD Uro-A—UM-A: 4.7 ± 1.0 nM; UM-B: 3.3 ± 2.6 nM IsoUro-A—UM-A: <LOD; UM-B: 2.7 ± 1.4 nM Uro-B—UM-A: <LOD; UM-B: 4.3 nM
			*Urine samples:* 0.1% formic acid in H_2_O	HPLC-DAD-ESI-Q-MS	*Trial 1:* UM-A (57%): 28.3 ± 12.1 nM; UM-B (43%): 28.8 ± 14.6 nM; UM-0: 0% *Trial 2:* UM-A: 44%, UM-B: 55%; UM-0: 0%
Clinical [42]	RCT, parallel: 284 healthy (33 females), age: 51.1 ± 10.6 y	WK—28 g/day, 18 months MED diet (+440 mg polyphenols/day) Control: HDG diet	*Blood and urine samples:* 12 h fast, baseline, 18 months	HPLC-QTOF Q-TOF-LC/MS	*Urine:* Uro-A—NQ
Clinical [45]	CT: 52 PD patients (21 females), age: 68 ± 8 y (44–88) 117 healthy (48 females), age: 60 ± 6 y (44–72)	WK—30 g/day, 3 days	*Urine samples:* 0.1% formic acid in H_2_O	UPLC-ESI-QTOF-MS	PD patients: UM-A: 45%, UM-B: 27.5%, UM-0: 27.5% Healthy control: UM-A: 57%, UM-B: 34%, UM-0: 9%
Clinical [46]	RCT, parallel: 286 obese participants (34 females), BMI = 31.2 kg/m^2^ VAT = 29%, age: 50.8 ± 10.4 y	WK—28 g/day, 18 months MED diet (+440 mg polyphenols/day) Control: HDG diet	*Blood and urine samples:* 12 h fast, baseline, 18 months	HPLC-QTOF Q-TOF-LC/MS	*Urine:* Uro-A—NQ
Clinical [49]	RCT, parallel: 256 healthy (28 females), age: 51.3±10.6 y	WK—28 g/day, 18 months MED diet (+440 mg polyphenols/day) Control: HDG diet	*Blood and urine samples:* 12 h fast, baseline, 18 months	HPLC-QTOF Q-TOF-LC/MS	*Urine:* Uro-A—NQ Uro-C—NQ

*^a^* quantified with UPLC-QQQ; *^b^* quantified with HPLC-DAD; ACN—acetonitrile; BMI—body mass index; BPH—benign prostatic hyperplasia; CT—controlled trial; DIRECT PLUS—Dietary Intervention Randomized Controlled Trial Polyphenols Unprocessed Study; DMEA—dimethyl ellagic acid; DWP—defatted walnut powder; DWPE—defatted walnut powder extract; gluc—glucuronide; green-MED diet—Mediterranean diet higher in polyphenols and lower in red/processed meat; HC—healthy control; HDG—healthy dietary guidelines; HOC—hippocampal occupancy score; IsoUro-A—isourolithin A (3,9-dihydroxy-urolithin); LOD—limit of detection; LOQ—limit of quantification; LS mean—least square means; MED diet—Mediterranean diet; ND—not detected; NQ—not quantified; PCa—prostate cancer; PD—Parkinson’s disease; PWK—peeled walnut kernel; RCT—randomized controlled trial; Tr—traces amount; UM-0—urolithin non-producers; UM-A—metabotype A (only urolithin A producers); UM-B—metabotype B (urolithin A, isourolithin A and urolithin B producers); UMs—urolithin metabotypes; Uro-A—urolithin A (3,8-dihydroxy urolithin); Uro-B—urolithin B (3-hydroxy-urolithin); Uro-C—urolithin C (3,8,9-trihydroxy-urolithin); Uro-D—urolithin D (3,4,8,9-tetrahydroxy-urolithin); Uro-M6—urolithin M6 (3,8,9,10-tetrahydroxy-urolithin); Uro-M7—urolithin M7 (3,8,10-trihydroxy-urolithin); VAT—visceral adipose tissue; WK—walnut kernel; WKOA—walnut kernel organic acid; WKP—walnut kernel protein; WKPS—walnut kernel polysaccharides; WO—walnut kernel oil.

## Data Availability

Data are contained within the article.

## Abbreviations

AA—antioxidant activity; ABTS—2,2′-azino-bis(3-ethylbenzothiazoline-6-sulfonic acid); AChE—acetylcholinesterase; AD—Alzheimer’s disease; AGEs—advanced glycation end products; AHPA—acid-hydrolysable phenolic acid; AI—anti-inflammatory; ApoA-I—apolipoprotein A-I; ApoB—apolipoprotein B; BChE—butyrylcholinesterase; BDNF—brain-derived neurotrophic factor; BMI—body mass index; BPA—bound phenolic acid; BPH—benign prostatic hyperplasia; CA2—carbonic anhydrase 2; CAT—catalase; CRC—colorectal cancer; CE—cold extract; CE-MS—capillary electrophoresis mass spectrometry; ChAT—choline acetyltransferase; CMD—cardiometabolic disorders; CMR—cardiometabolic risk; CPS1—carbamoylphosphate synthetase; CREB—cAMP-response element binding protein; CRF—cardiometabolic risk factors; CSCs—cancer stem cells; CT—clinical trial; CTC—condensed tannin content; cv.—cultivar; DAD—diode array detector; DCF-DA—2′,7′-dichlorodihydrofluorecein diacetate; D-gal—D-galactose; DIRECT PLUS—Dietary Intervention Randomized Controlled Trial Polyphenols Unprocessed Study; DMEA—dimethyl ellagic acid; DPPH—2,2-diphenyl-1-picrylhydrazyl; dw—dry weight; DWP—defatted walnut powder; DWKP—defatted walnut kernel with pellicle; DWPE—defatted walnut powder extract; EA—ellagic acid; EAC—ellagic acid content; ECG—epicatechin gallate; EGCG—epigallocatechin gallate; ESI—electrospray ionization; ETC—ellagitannin content; EtOH—ethanol; ETs—ellagitannins; FD—fluorescence detector; FPA—free phenolic acid; FRAP—ferric reducing antioxidant power; fw—fresh weight; GA—gallic acid; GAE—gallic acid equivalents; GC—Gim-cheon 1ho cultivar; GC/MS—gas chromatography–mass spectrometry; GCG—gallocatechin gallate; Gla A—Glansreginin A; Gla B—Glansreginin B; Gla C—Glansreginin C; Gluc—glucuronide; GM—gut microbiota; GnM—gene modulation; green-MED diet—Mediterranean diet higher in polyphenols and lower in red/processed meat; HAEC—human aorta endothelial cells; HC—healthy control; HDG—healthy dietary guidelines; HDL-c—high-density lipoprotein cholesterol; HE—heat extract; HFD—high-fat diet; HHDP—hexahydroxydiphenic acid; HOC—hippocampal occupancy score; HOMA-IR—Homeostatic Model Assessment for Insulin Resistance; HPIC—high-performance ion chromatography; HPLC—high-performance liquid chromatography; HR—high resolution; ICAM-1—intracellular adhesion molecule 1; IDL-c—intermediate lipoprotein cholesterol; i.g.—intragastrically; IL—interleukin; IMS—ion mobility separation; INF-γ—interferon gamma; LC—liquid chromatography; LDH—lactate dehydrogenase; LDL-c—low-density lipoprotein cholesterol; LOLA—L-ornitine L-aspartate; LPS—lipopolysaccharide; LTQ—hybrid linear trap quadrupole; LVVc—lateral ventricle volume; mAge—methylation-assessed biological age; MC—4-methyl-catechol; MDA—malondialdehyde; MED diet—Mediterranean diet; MeOH—methanol; MetS—metabolic syndrome; MG—model group; MMP—mitochondrial membrane potential; MS—mass spectrometry; MSn—multi-stage mass spectrometry; MTT—3-(4,5-dimethylthiazol-2-yl)-2,5-diphenyltetrazolium bromide; MWM—Morris Water Maze test; NAFLD—non-alcoholic fatty liver disease; NF-κB—nuclear factor kappa-light-chain-enhancer of activated B cells; NASH—non-alcoholic steatohepatitis; NMR—nuclear magnetic resonance; NQ—non-quantified; ORAC—oxygen radical absorbance capacity; PBMCs—peripheral blood mononuclear cells; PCa—prostate cancer; PCL—photochemiluminesence; PD—Parkinson’s disease; PDA—photodiode array detector; PHA—phytohemagglutin; PKA—protein kinase A; PMA—phorbol myristate acetate; PWK—peeled walnut kernel; PWKE—peeled walnut kernel extract; QqQ-MS—triple quadrupole mass spectrometry; qPCRs—specific real-time quantitative polymerase chain reactions; Q-TOF—quadrupole time of flight; RCT—randomized controlled trial; RNS—reactive nitrogen species; ROS—reactive oxygen species; RRFs—relative response factors; RT-PCR—reverse transcriptase polymerase chain reaction; SCFA—short-chain fatty acids; SOD—superoxide dismutase; TA—tannic acid; TAPC—total analyzed phenolics content; T2D—type 2 diabetes; TE—Trolox equivalents; TFC—total flavonoid content; TG—triglycerides; TLC—thin-layer chromatography; TNFα—tumor necrosis factor-α; T-AOC—total antioxidant capacity; TPC—total phenolic content; UM—urolithin metabotypes; UPLC—ultra-performance liquid chromatography; Uros—urolithins; vars.—varieties; VAT—visceral adipose tissue; VCAM-1—vascular cell adhesion molecule 1; VLDL-c—very-low-density lipoprotein cholesterol; WAT—white adipose tissue; WE—walnut extract; WK—walnut kernel; WKE—walnut kernel extract; WKH—walnut kernel hydrolysate; WKOA—walnut kernel organic acid; WKP—walnut kernel protein; WKPhs—walnut kernel polyphenols; WKPSs—walnut kernel polysaccharides; WO—walnut kernel oil; WP—walnut pellicle; WPhs—walnut polyphenols; WPE—walnut pellicle extract; WPhE—walnut phenolic extract; WS—walnut skin.
